# Interlacing biology and engineering: An introduction to textiles and their application in tissue engineering

**DOI:** 10.1016/j.mtbio.2025.101617

**Published:** 2025-02-25

**Authors:** S. Scholpp, L.A. Hoffmann, E. Schätzlein, T. Gries, C. Emonts, A. Blaeser

**Affiliations:** a Institute for BioMedical Printing Technology, Technical University of Darmstadt, Darmstadt, Germany; b Institut für Textiltechnik, RWTH Aachen University, Aachen, Germany; c Centre for Synthetic Biology, Technical University of Darmstadt, Darmstadt, Germany

**Keywords:** Biofabrication, Regenerative medicine, Tissue engineering, Textiles, Scaffolds, Fibers

## Abstract

Tissue engineering (TE) aims to provide personalized solutions for tissue loss caused by trauma, tumors, or congenital defects. While traditional methods like autologous and homologous tissue transplants face challenges such as donor shortages and risk of donor site morbidity, TE provides a viable alternative using scaffolds, cells, and biologically active molecules. Textiles represent a promising scaffold option for both *in-vitro* and *in-situ* TE applications.

Textile engineering is a broad field and can be divided into fiber-based textiles and yarn-based textiles. In fiber-based textiles the textile fabric is produced in the same step as the fibers (e.g. non-wovens, electrospun mats and 3D-printed). For yarn-based textiles, yarns are produced from fibers or filaments first and then, a textile fabric is produced (e.g. woven, weft-knitted, warp-knitted and braided fabrics).

The selection of textile scaffold technology depends on the target tissue, mechanical requirements, and fabrication methods, with each approach offering distinct advantages. Braided scaffolds, with their high tensile strength, are ideal for load-bearing tissues like tendons and ligaments, while their ability to form stable hollow lumens makes them suitable for vascular applications. Weaving, weft-, and warp-knitting provide tunable structural properties, with warp-knitting offering the greatest design flexibility. Spacer fabrics enable complex 3D architecture, benefiting applications such as skin grafts and multilayered tissues. Electrospinning, though highly effective in mimicking the ECM, is structurally limited. The complex interactions between materials, fiber properties, and textile technologies allows for scaffolds with a wide range of morphological and mechanical characteristics (e.g., tensile strength of woven textiles ranging from 0.64 to 180.4 N/mm^2^). With in-depth knowledge, textiles can be tailored to obtain specific mechanical properties as accurately as possible and aid the formation of functional tissue. However, as textile structures inherently differ from biological tissues, careful optimization is required to enhance cell behavior, mechanical performance, and clinical applicability.

This review is intended for TE experts interested in using textiles as scaffolds and provides a detailed analysis of the available options, their characteristics and known applications. For this, first the major fiber formation methods are introduced, then subsequent used automated textile technologies are presented, highlighting their strengths and limitations. Finally, we analyze how these textile and fiber structures are utilized in TE, organized by the use of textiles in TE across major organ systems, including the nervous, skin, cardiovascular, respiratory, urinary, digestive, and musculoskeletal systems.

## Graphical abstract

**Figure:**
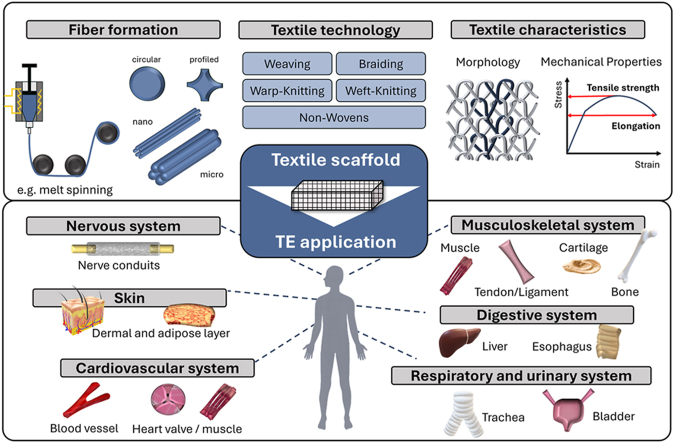

## 1 Introduction

Trauma, tumor-related complications, or congenital influences can lead to the loss of functional body tissue such as muscles, tendons, and skin. The gold standard for treatment is autologous or homologous tissue transplantations [1,2]. Autologous transplants use the patient's own tissue [3], with the risk of donor site morbidity [4,5], while homologous transplants, using genetically different donors of the same species [6,7], are limited by donor availability, especially for complex organs [8,9].

Tissue engineering (TE), addresses these problems by aiming to restore or replace damaged tissue and organs [[10], [11], [12]]. Although tissue engineering has already shown initial success, particularly in bone [[13], [14], [15]] and vascular implants [[16], [17], [18]], the production of flexible, large tissues such as the heart remains a major challenge.

Given the fibrous nature of the extracellular matrix (ECM) in biological tissue, the use of fibrous textiles that provide cell alignment through contact guidance and sufficient stability for implantation (e.g. suture retention) is a logical approach for the development of scaffold-based tissue constructs [19]. In recent years, textile structures have been increasingly used in biomedical implants to support or replace both soft and hard tissues [20]. Thanks to their adaptable structures and strong mechanical and biological properties, fibrous scaffolds are particularly suitable for mimicking the architecture and mechanical properties of natural tissues [19,21].

Although the biological and medical perspective is often highlighted in TE reviews, the inherent mechanical and morphological properties of the different fibers and textiles is seldomly shown. Textiles can be engineered to fit certain properties, but the selection of material, fiber formation technique and textile production technique is often challenging. In addition, each textile technology offers a variety of design freedoms. In the jungle of textile options, the question of which textile to use for one specific application stands out. This review aims to provide insight into this question from a textile technology point of view.

In this review, the role of textiles in tissue engineering is studied, starting with an overview of key methods, techniques, and requirements in the field of tissue engineering. In the second part of this review, the common fiber formation methods as well as the main yarn-based and fiber-based textile structures are introduced, including main options for textile design choices. Finally, approaches for textile scaffolds are analyzed for different target tissues. The textile and it's fit for demonstrated application are discussed. This review is intended for TE experts interested in using textiles as scaffolds, providing a detailed analysis of the available options, their characteristics and known applications.

## 2 Tissue engineering methods, techniques and requirements

**Fig. 1:**
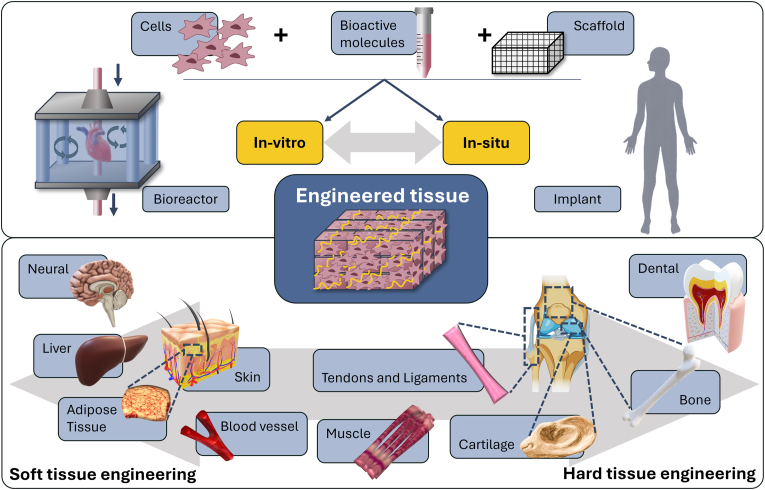
*In-vitro* and *in-situ* methods that lead to soft or hard tissue engineering. *In-vitro,* the building blocks (cells, bioactive molecules and scaffolds) are combined and cultivated in e.g., bioreactors before implantation. *In-situ*, the building blocks are implanted to the damaged area, to provide mechanical support and to develop new tissue at the target site. The engineered tissue can then be categorized as soft (e.g., neural tissue) or hard (e.g., bone tissue) depending on the application.

In tissue engineering, multiple methods have evolved. These methods can be classified broadly into two main categories: *In-vitro* and *in-situ* TE [22]. Each approach utilizes different techniques to achieve tissue regeneration. Due to the range of mechanical properties of human tissue, approaches can be further divided into soft or hard tissue engineering (Fig. 1).

*In-vitro* TE involves the construction of tissues outside the body under specific laboratory conditions. This typically includes isolating cells, culturing them, and constructing tissue-like structures using biomaterials, growth factors, and mechanical stimulation [22,23]. In *in-situ* TE, cells, growth factors or scaffolds are implanted directly into the body to provide mechanical support and guide the natural healing processes. This allows new tissue to develop at the target site without pre-cultivation [[24], [25], [26]].

Common TE techniques include molding, cell seeding and 3D bioprinting. In molding, a cell-laden hydrogel is cast into molds, crosslinks and forms three-dimensional tissue-like structures [27,28]. In cell seeding, cells are seeded onto a scaffold, with factors such as seeding density, spatial distribution and scaffold characteristics influencing cell growth and tissue formation [[29], [30], [31]]. To improve the spatial distribution of cells, 3D bioprinting combines cells with a hydrogel matrix, which is then deposited in droplets, lines, or layers to fabricate a predefined three-dimensional structure [[32], [33], [34]]. Bioprinting enables a precise spatial arrangement of cells and materials [[35], [36], [37]].

Soft tissue engineering focuses on the regeneration of soft tissues such as skin, muscle and fat, often addressing injuries or defects caused by trauma, disease, or aging [38,39]. These tissues are typically elastic and non-mineralized, and scaffolds require flexible materials with lower stiffness to mimic the properties of native soft tissue (Table 1). Therefore, polymers and especially hydrogels are often used that support the adhesion and growth of cells as well as the mechanical flexibility [[40], [41], [42]]. In contrast, hard tissue engineering focuses on the regeneration and repair of hard tissues such as bone and teeth, addressing injuries or defects caused by fractures, bone diseases, or dental issues [38,43,44]. Hard tissues are rigid and mineralized, so materials like bio-glass [45,46], ceramics [44,47], polymers [40,48] and metals [49,50] are often used due to their mechanical properties.

**Table 1:** Excerpt of the mechanical properties of human tissue and collagen I hydrogels in concentration ranges commonly used in 3D cell culture (0.3–6 mg/ml).

Tissue	Tensile strength [N/mm^2^]	Compressive strength [N/mm^2^]	Young's modulus [N/mm^2^]	Shear modulus [N/mm^2^]	Ref.
**Collagen I**	0.008-0.774	0.77-42.8 × 10^−3^	0.008-7.19	1.71–1440 × 10^−6^	[60,[76], [77], [78]]
**Cancellous bone**	1–5	2–41	50–5000	72–499	[[79], [80], [81], [82], [83]]
**Cortical bone**	60–193	100–230	3000–30000	3300-6000	[79,80,[83], [84], [85], [86], [87]]
**Cartilage**	3.7–40	1.16–7.75	0.7–15.3	1–1.5	[79,[88], [89], [90]]
**Ligaments**	13–46	–	25–4300	0.004-0.88	[79,84,[91], [92], [93], [94]]
**Tendons**	24–140	–	143–2310	0.036-1.6	[79,91,92,[95], [96], [97]]
**Blood vessels**	0.03–4	–	0.1–130	–	[[98], [99], [100]]
**Heart muscle**	3 -15 × 10^−3^	–	0.02-0.50	–	[[101], [102], [103]]
**Skeletal muscle**	0.01-0.02	–	0.01-0.45	0.023-0.033	[91,102,[104], [105], [106]]
**Skin**	3–14	–	0.05–160	0.001–3.1	[84,91,[107], [108], [109]]

Hydrogels play an important role in both soft and hard tissue engineering. They support 3D cell culture by mimicking the water content and structure of the ECM [51,52]. Common types, like collagen and fibrin [33,53,54], aid cell growth but lack the mechanical strength needed for e.g. load-bearing tissues [[55], [56], [57]]. Higher concentrations can increase stiffness, but often compromise the biological compatibility and properties needed for effective cell culture, creating a trade-off [[58], [59], [60], [61]].

To overcome this, soft hydrogels are reinforced with additional scaffolds [[62], [63], [64]]. The properties of the scaffold, such as porosity and stiffness, are of critical importance as they can direct stem cells differentiation (e.g., stiffer scaffolds for chondrogenic differentiation) [65]. The porosity (pore size and distribution) of scaffolds is crucial for tissue formation because it enables cell nutrition, proliferation, migration, tissue vascularization, and new tissue formation [66,67]. Larger pores (160–270 μm) facilitate the growth of blood vessels [68], while medium sized pores (250–500 μm) promote chondrocyte proliferation and ECM production [69]. Highly porous scaffolds support cell growth and migration but cannot provide the strength required for implantation and surgical handling [66,67,70]. A balance between porosity and mechanical properties is critical.

These scaffolds are commonly fabricated with additive manufacturing technologies such as fused filament fabrication or selective laser melting [43,47,71]. However, textiles offer a customizable scaffold option, with the size and arrangement of fibers influencing cell orientation and behavior [[72], [73], [74], [75]]. By customizing textile scaffolds, specific cell interactions can be supported, making them an interesting choice to produce scaffolds that support the desired cell behavior and tissue development.

## 3 Textile production

**Fig. 2:**
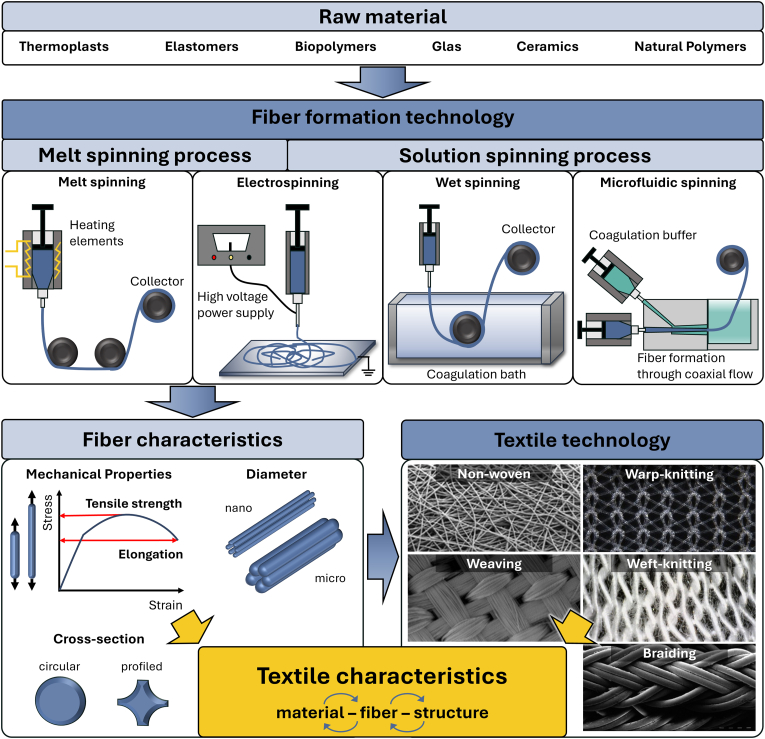
Textile characteristics depend on the used material, the fiber formation technology with the resulting characteristics, as well as on the textile technology and their resulting structural characteristics. Examples of raw materials and overview of fiber formation techniques used in tissue engineering applications: In melt spinning, molten polymer is extruded through a spinneret to form solid fibers; in electrospinning, viscoelastic polymer fibers are drawn through an electric field between a needle and a collector plate; in wet spinning, fibers are produced by injecting a polymer solution into a coagulation bath for crosslinking; in microfluidic spinning, fibers are produced by coaxial flow of prepolymer and crosslinking agent. This results in fiber characteristics (diameter, cross-section and mechanical properties) that are dependent on the chosen technique and the raw material. The final textile properties then also depend on the textile technology used, which leads to different structures (examples of common structures are shown) (Adapted from Refs. [72,117,118,127,128]).

A textile is a flexible material made of an interlacing network of fibers [110,111]. Textiles can be grouped into fiber-based and yarn-based textiles. For fiber-based textiles the fabric is produced in the same step as the fibers. Examples are different non-wovens and electrospun mats. In contrast, yarn-based textiles require an additional step: fibers or filaments are first spun into yarns, which are then used to create fabrics such as woven, weft-knitted, warp-knitted or braided fabrics (Fig. 2).

### 3.1 Fiber formation technology

Material selection is critical for textile-based tissue engineering. The material's properties, including its biocompatibility, cell adhesion, and mechanical characteristics, are determined by this process. For instance, rigid materials are suitable for bone scaffolds, while flexible materials are better suited for vascular grafts. Biocompatibility is of crucial importance and can be assessed through *in-vitro* cytotoxicity tests (e.g., ISO 10993–5:2009, MTT, LDH) to ensure materials support tissue regeneration [112]. The material that is to be spun, as well as the intended morphological and mechanical properties are decisive criteria for technology selection. Beyond biological compatibility, materials must meet manufacturing requirements, such as sufficient strength to prevent tearing during fiber and textile formation. Suitable materials for TE include thermoplastics, elastomers, biopolymers, glass, ceramics, and natural polymers (Fig. 2). Depending on the material, only certain fiber formation technologies are applicable. The choice of material and fiber formation technology determines the characteristics and mechanical properties of the fiber. Polymers can be spun into fibers with varying characteristics, including fiber diameter and cross-sectional shape, as monofilament or multifilament yarns. In combination with the textile structure, which in turn depends on the textile technology used, the final characteristics of the textile are defined [[113], [114], [115], [116]].

The most common and upscaled fiber formation technologies are melt spinning and solution spinning. Additionally, small scale fiber production technologies such as electrospinning and microfluidic spinning are in use. In depth explanations of fiber formation technologies can be found in Veit [115], Fourné [114] and Eichhorn et al. [113].

The basic process of fiber spinning begins by transferring a solid polymer into a liquid state. The source of polymers can be classified as synthetic (e.g. thermoplasts, elastomers, biopolymers) or natural (e.g. natural silk). In melt spinning this is done by melting, while in solution spinning a solvent is used. The liquid polymer then passes through a nozzle, followed by a drawing unit to align the macromolecules in a rubbery state in the direction of the material flow. Before the fibers are wound and collected on a spool, they are either cooled or the solvent is removed, depending on the process. Simplified, the viscosity and rheology of the polymer, both in the liquid and solid state, control the processability of the fiber, while the spinning process shapes the form of the fiber and the structure of the fiber is mainly set by the drawing process [[113], [114], [115], [116], [117], [118]]. Melt spinning is a preferred method for processing thermoplastic polymers due to its cost-effectiveness, simplicity and especially environmental friendliness, because it does not require solvents [119,120]. Common polymers for TE applications are polyamide (PA), polylactide (PLA), polyetheretherketone (PEEK) and polycaprolactone (PCL) [116,121]. For detailed explanation, see Hufenus et al. [121].

In solution spinning the polymer material is dissolved in a solvent and drawn out of a spinneret. The solvent then needs to be removed from the fiber either in a dry surrounding (dry solution spinning) or a wet bath (wet solution spinning/wet spinning) [[113], [114], [115]]. This technology enables the production of fibers with large pore sizes and high porosity. Limitations of the solution spinning process are the solubility of polymers and possible solvent residues in the fiber. Common polymers for TE applications are PCL, PLA, silk fibroin (SF) or collagen [116,122,123].

Microfluidic spinning is a special form of solution spinning in which a microfluidic channel is used with a coaxial flow of the prepolymer solution and a crosslinking agent that causes on-demand polymerization. Depending on the flow rate of the core and shell solution, the channel dimensions and the viscosity of the solutions, the diameters of the fibers can be adjusted and the fiber is drawn [123,124].

Electrospinning is a technology used to produce nanofibers. Either a molten or a dissolved polymer fiber is drawn from a drop of polymer solution at the spinneret. Using an electric field applied between the injecting needle and a collector, the fiber is stretched and deposited randomly on the collector. It offers effective control over the fiber design by setting key process parameters like voltage and flow rate. The resulting nanofibers can be processed to nanofiber yarns on a small scale [125,126].

### 3.2 Textile technologies for scaffold fabrication

Traditional textile production methods are the yarn-based textile technologies weaving, braiding, weft-knitting and warp-knitting. Yarns are connected with each other through undulation (weaving and braiding) or stitch formation (weft- and warp-knitting). In addition, the relatively new fiber-based textile technologies electrospinning and melt electro writing are often used for the direct fabrication of scaffolds from polymer or polymer solution [127,[129], [130], [131], [132]]. In depth explanations of textile technologies can be found in Kyosev and Boussu [133] for weaving, Spencer [134] and Weber and Weber [135] for weft- and warp-knitting, Raz [136] for warp-knitting, and for braiding see Kyosev [137]. An overview of the presented textile technologies with qualitative information on key mechanical properties and production speed is given in Table 2.

**Table 2:** Overview of presented textile technologies with qualitative information on key mechanical properties and production speed.

Textile Technology	Structure	Binding element	Mech. strength	Mechanical stretch	Drapability	Fiber Diameter	Production speed
**Weaving**	Flat, Tubular, Voluminous	Interlaces	High	Low	Medium	μm to mm	High (up to 1000 wefts/min^2^)
**Braiding**	Flat, Tubular	Interlacing	High	Low	High	μm to mm	High (up to 58 rounds/min of carrier^3^)
**Weft-knitting**	Flat, Tubular, Voluminous	Stitches	Low	High	High	μm to mm	High (up to 40 rounds/min^4^)
**Warp-knitting**	Flat, Tubular, Voluminous	Stitches	Medium	Highly variable	High	μm to mm	High (up to 4400 wales/min [170])
**Electro-spinning**	Flat, Tubular, Curved	–	Low	Low	Medium	nm to μm	Low (up to 5 m/min^5^)
**Melt Electro Writing**	Flat, Voluminous	–	Low	Low	Low	nm to μm	Low (not commercially available)

#### 3.2.1 Weaving

**Fig. 3:**
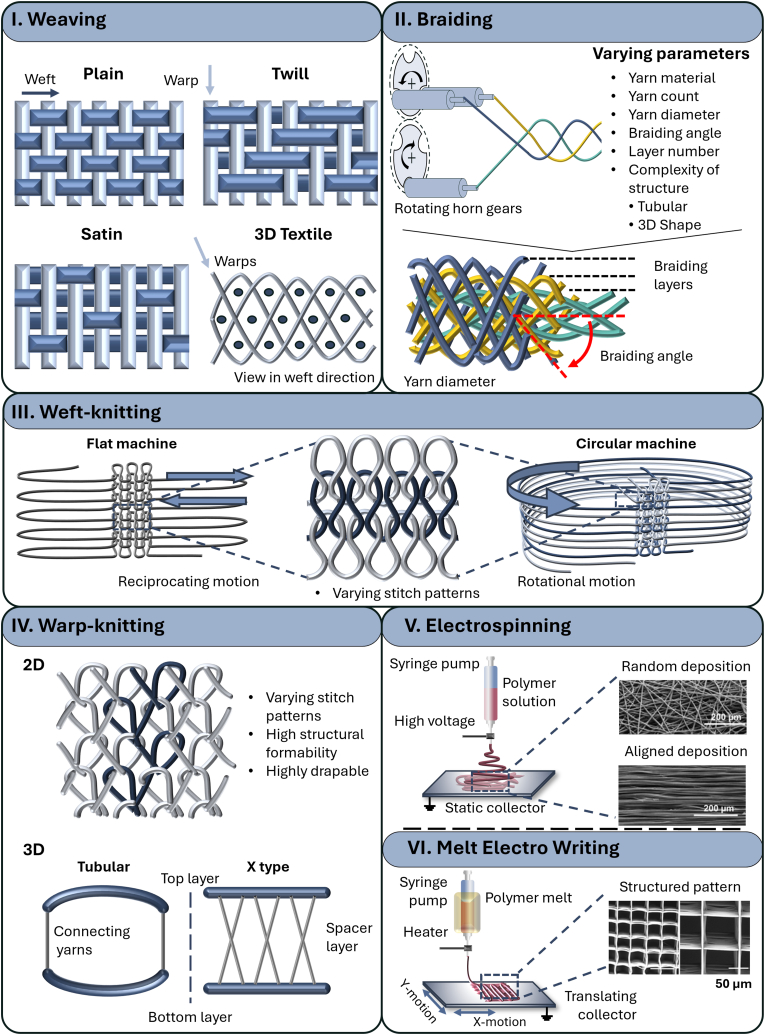
**I.** Weaving works by interlacing weft and warp fibers in a vertical direction. 2D structures: Top view of the most common weave patterns: plain weave, twill weave and satin weave. The dark stands for the weft fibers and the light for the warp fibers. 3D Structures: View in weft direction for one pattern (Adapted from Ref. [129]). **II.** Braiding works by interweaving yarns in an angular direction (10°–80°) to each other. Interesting parameters are yarn count, diameter, braiding angle and number of layers (Adapted from Refs. [141,171]). **III.** Weft-knitting works by drawing fibers horizontally through a previous loop to create a series of connected loops (either on a flat or circular machine). **IV.** Warp-knitting works by drawing fibers vertically through a previous loop to create a series of connected loops. By adding connecting yarns on the side of two layers tubular structures can be created, and by adding a full layer, so called spacer fabrics are created. **V/VI.** Schematic drawing of electrospinning (left) and melt electro writing (right), both use an applied electric field between an injecting needle and a collector plate or cylinder in a distance to the needle to drawn fibers (adapted from Ref. [172]). Electrospinning can result in random or aligned microfibers and can be combined into a hybrid scaffold with e.g. a knitted fabric [159]. Melt electro writing as a special form of electrospinning can result in structured patterns similar to 3D printing [167].

In weaving, the textile is formed by interlacing a weft yarn with warp yarns at a 90° angle. The warp yarns run in the longitudinal direction of the textile while the weft yarn runs in the transverse direction. The three most common weaves are plain weave, twill weave, and satin weave. They differ in the pattern of undulation of the weft and warp system (Fig. 3 I). Beside 2D flat textiles, 3D structures can be woven in tubular form or produced by interweaving several weft yarn layers with warp yarns [127,133].

The weaving technology can be used with a wide variety of yarn materials, e.g. PLA, Collagen, PET, PCL or silk fibroin [72,109,117,138]. Material as well as patterning and machine configuration affect the characteristics of the resulting textile. In general, weaving technology favors high mechanical strength in the direction of the yarns (longitudinal and transverse). More flexibility is common in diagonal direction. The flexibility and smoothness of the textile can be altered by using and combining different weaving patterns (e.g. plain, satin or twill weave). Plain weave, for example, where the weft and warp run alternately over each other, results in a dense structure with a maximum number of binding points. In comparison, satin weave is known for its smooth surface and less interlacing, as it has fewer binding points and longer floats. Therefore, satin is more flexible and tends to have more space between the yarns, making it more porous. Twill is also flexible however, it has a diagonal rib pattern due to the way the yarns are woven, which can make it less porous than satin fabric. It can provide a good balance between flexibility and strength.

In addition to the pattern, the number of yarns also influences the porosity and looseness of the fabric. Furthermore, the selected yarn material and yarn fineness also have significant influence on the mechanical properties [72,127,131,133,139].

#### 3.2.2 Braiding

A braided textile is made by interlacing a set of yarns in an angular direction (10°–80°) to each other (Fig. 3 II). Braiding can be used to form solid and hollow structures with different cross-sectional geometries e.g. tubular and hexagonal. Braided structures can be fabricated in 2D or 3D and hierarchically arranged. These structures offer high tensile strength, especially in axial direction, making them suitable candidates for tissue engineering of tendons and ligaments [137,140,141].

The most important parameters are the yarn material, yarn count, yarn diameter, the number of braiding layers and the complexity of the structure. A wide variety of polymers can be braided, such as PCL, PLA, PGA and PET [72,140,142]. The braiding angle is defined by the angle between the braided yarns and the longitudinal direction. It influences mechanical properties such as stiffness and porosity. The yarn diameter has an influence on the porosity and tensile strength of the structure. The number of braiding layers increases the three-dimensionality and as the number of layers increases, the scaffold becomes more stable with decreasing porosity [117,129,131,137,142,143].

#### 3.2.3 Weft-knitting

Weft-knitting is a stitch forming textile fabrication method (Fig. 3 III). A weft yarn is drawn through a previous loop creating a row of stitches. Stitches in one row are formed one after the other from the same yarn. This yarn can either run in a reciprocating motion or in a rotating motion, resulting in flat or tubular weft-knits [134,135].

In weft-knitting, pattern, the material and stitch densities significantly influence the fabric's properties. Choosing more open knit structures by skipping stitches in one row can increase porosity by creating additional spaces within the fabric [134,135]. Weft-knits have a high structural deformability and are therefore highly drapable. In combination with elastic yarns, this property is often used when elasticity and a form fit are needed, e.g. for skin [144] or blood vessels [145]. Common polymers used for scaffolds include PLGA, collagen, PET, PLA, SF, PCL, and PVA [72,117,145]. In addition, lower density caused by fewer needles per inch results in a looser, more open fabric with greater porosity. Finally, less tension on the yarn during knitting creates larger stitches, which again improves the porosity, flexibility, stretch and drape of the fabric [134,135,146]. The design of three-dimensional weft-knits gives the freedom to pattern two separate knitted layers and their connecting points [134,135].

#### 3.2.4 Warp-knitting

Warp-knitting is also a stitch forming textile fabrication method. However, in warp-knitting, stitches of one row are formed simultaneously, each stitch from a separate warp yarn. The textile is formed by connecting the warp yarn with the neighboring warp yarns during the stitch formation (Fig. 3 IV) [[134], [135], [136]].

Warp-knitting gives a great variability of patterning options. Combined with the yarn material(s) and stitch densities, a wide variety of structures and characteristics can be achieved such as 2D or 3D as well as loose or dense fabrics. However, warp-knitting is very complex and requires in-depth knowledge to create optimal structures [127,134,147].

By varying the pattern to a more open structure, porosity is increased (depending on the needle and bar configuration). Warp knitted fabrics offer high structural formability and are highly drapable, which can be further enhanced by using elastic yarns. Polymers commonly used for scaffolds include PLGA, PET, PLA, SF and PCL [72,117,145]. Like weft-knitting, reducing the tension in warp knitting can result in a more flexible and porous fabric. Lower tension creates larger stitches and more space between the threads, which contributes to a softer and more drapable fabric [[147], [148], [149]]. Compared to weft-knitting, warp-knitting machines are generally faster and produce fabrics that are more dimensionally stable, i.e. less prone to stretching and more resistant to running stitches [[134], [135], [136]].

Three-dimensional warp-knits consist of three yarn systems that can be patterned mostly independently. In tissue engineering, elasticity, bursting strength, porosity and pore size are often of interest. Due to the more adjustable mechanical characteristics, high porosity, high volume-to-weight ratio, moisture permeability, compressive strength and resilience, it offers great potential as scaffold material in TE applications. For example, depending on structure and material, the height ranges typically between 100 μm and 60 mm [72,127,150].

#### 3.2.5 Electrospinning and melt electro writing

Electrospinning has emerged as an interesting and promising method to directly fabricate scaffolds. Electrospinning generates nanofiber mats using the solution spinning process (Section 3.1). The filament is deposited randomly on a collector which can be flat creating nanofiber mats, or round and turning creating nanofiber tubes with aligned filaments (Fig. 3 V). These nanofiber structures can be used as porous scaffolds with suitable mechanical properties for tissue engineering applications [117,127], such as nerve conduits applications [151], or skin substitutes applications [152]. However, with decreasing fiber diameter and increasing overlap, the pore sizes of these fiber mats can be too small (<100 μm) to allow successful cell infiltration by e.g. fibroblast cells (size 10–100 μm), meaning that the cells can only grow on the surface of these mats. Nevertheless, this is particularly important for implants that should successfully grow into surrounding tissue [[153], [154], [155], [156], [157]]. Additionally, electrospinning is sometimes combined with textiles made from weaving [158], knitting [[159], [160], [161]] or braiding [[162], [163], [164], [165]]. By combining the high surface area and density of electrospun nanofiber mats with the structural strength and flexibility of conventional textiles, composite structures with advanced properties such as selective permeability can be created.

Melt electro writing is similar to electrospinning however the fibers can be placed with more precision in the horizontal direction. Therefore, it bridges the gap between electrospinning and fused filament fabrication additive manufacturing processes, thus can be categorized as a special form of 3D printing (Fig. 3 VI). In melt electro writing the polymer is spun in a melt spinning process and drawn to a fiber by the applied electric field. This electric field ensures a uniform polymer jet and plays a significant role by regulating important properties such as the flight path and fiber diameter of the polymer jet. In contrast to electrospinning, either the collector or the syringe is moved, which enables the fabrication of precisely controlled and reproducible 3D fiber structures with high accuracy and a maximum height of 10 mm [166]. Melt electro writing has the capability of fabricating controllable and highly ordered complex geometries from a wide range of polymers such as PLA, PBS, PLLA and PCL [132,[167], [168], [169]].

## 4 Applications of textile scaffolds in tissue engineering

Textiles have developed into interesting scaffolds for tissue engineering. They offer structural properties such as high porosity, adjustable strength and materials that support cell growth and tissue regeneration. Their versatility enables the development of scaffolds tailored to specific organ systems, making them valuable for the repair and regeneration of various tissues [72,117,118,127,130].

**Fig. 4:**
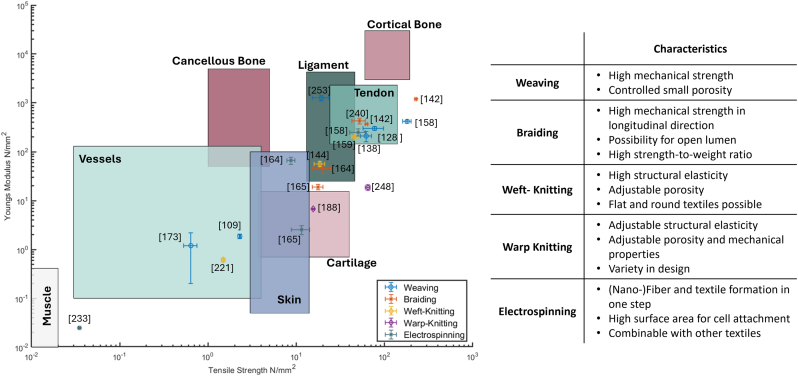
Review of mechanical properties of textile-based scaffolds for different target tissues. Colored Areas are mechanical property ranges of human tissue (Table 1). Summary of characteristics for weaving, braiding and weft-knitting, warp-knitting and electrospinning (Adapted from Ref. [129]).

However, the interactions between material, textile technology and textile design are very complex, resulting in a wide range of possible mechanical properties for each technology. Fig. 4 shows an excerpt of the investigated textile-based scaffolds in terms of their Young's modulus and tensile strength compared to the property ranges of human tissue (colored areas). The examples are classified according to the specific textile technology utilized.

The data illustrates the wide spectrum of mechanical properties exhibited by each technology. For instance, the tensile strength of woven scaffolds (blue datapoints) stretches over the range of 0.64 N/mm^2^ [173] to 180.4 N/mm^2^ [158]. However, if the target equivalent (Table 1) is compared with the properties achieved, these do not necessarily match. For example, research conducted by Liao et al. [173] aimed to replicate the properties of cartilage tissue, while that conducted by Shao et al. [158] aimed to replicate the properties of bone. Nevertheless, some research groups have achieved this, for example, Rothrauff et al. [164] developed a braided scaffold that closely mimics ligament tissue, while Wu et al. [138] created a woven scaffold tailored for tendons. Still, especially the extremes of soft and hard tissues such as muscles or bones remain a challenge. In conclusion, the complexity of the dependencies does not recommend a specific textile technology for a specific target tissue but rather requires in-depth knowledge and optimization.

This section explores the application of textile scaffolds in major organ systems (nervous, skin, cardiovascular, respiratory, urinary, digestive and musculoskeletal), reviewing not only their role in the development of tissue engineering solutions, but also the specific textile methods used in each example.

### 4.1 Nervous system

The nervous system poses a particular challenge for tissue engineering due to its complex structure and the need for precise guidance of regenerating nerve fibers. Textile scaffolds can offer interesting solutions, particularly through the use of tubular structures that can mimic the natural architecture of nerve pathways [[174], [175], [176]].

#### 4.1.1 Nerve conduit

**Fig. 5:**
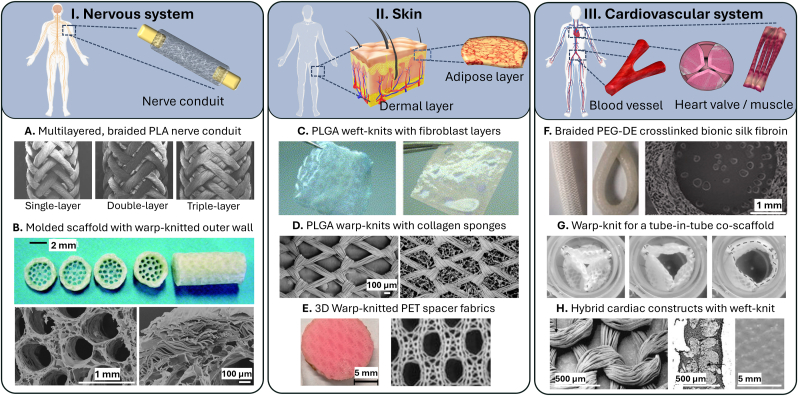
Overview of applications of textile-based scaffolds for tissue engineering in the nervous, skin, and cardiovascular system. A. multilayered, braided PLA nerve conduit [177], B. combined molded multichannel nerve guidance scaffold with a porous warp-knitted outer wall [178], C. dermal-like construct by combining 3D-knitted PLGA meshes with fibroblast layers [144,182], D hybrid warp-knitted PLGA scaffold with web-like collagen microsponges [183], E. 3D warp-knitted PET spacer fabrics successfully differentiated the hMSC into the adipogenic line [188], F. tubular, braided scaffold of PEG-DE cross-linked bionic silk fibroin for use in small-diameter vascular grafts [196], G. warp knitted fabric for implantation in the aortic position [147], H. hybrid cardiac constructs [202].

Tubular textile structures can be produced either directly or by winding flat textiles, allowing the creation of nerve conduits with customized properties. Lu et al. [177] showed that their multilayered, braided PLA nerve conduits can successfully bridge a 10 mm gap in the sciatic nerve of a rat, while the temporal and spatial progression of cellular activity within the conduit is comparable to biodegradable nerve conduits used in the literature (Fig. 5 A). Wang et al. [178] combined a molded multichannel nerve guidance scaffold with a porous warp-knitted outer wall, which resulted in improved mechanical properties and structures for cell migration and axonal elongation, ultimately supporting nerve regeneration in a natural fascicle form. In an in-vitro characterization, they demonstrated that the scaffolds have suitable mechanical strength, porosity, swelling properties and biodegradability (Fig. 5 B). Politikou et al. [179] performed digital nerve reconstruction with a silk fibroin-based textile in four human patients with a nerve defect greater than 5 mm. Their scaffold is a three-layer tubular construct. The middle layer is braided with silk fibroin fibers, and the inner and outer layers are electrospun silk fibroin. At 32 months, patients reported minimal to no pain and good sensory recovery. There was no evidence of inflammation or scarring.

While autologous nerve grafts remain the clinical standard for nerve repair, tissue-engineered nerve conduits offer a promising alternative. Textile-based conduits enable control over mechanical and structural properties, directly influencing the axonal regeneration. Braided scaffolds, as demonstrated by Lu et al. [177], provide high radial stability while maintaining an open lumen, a critical requirement for guided nerve regrowth. Although warp-knitted conduits are less frequently investigated, their integration with additional components can enhance their mechanical and biological performance [178]. The combination of electrospun nanofiber mats with other textile structures offers a promising approach that integrates the mechanical stability of braided scaffolds with the density of electrospun mats. Politikou et al. [179] demonstrated the clinical applicability of such hybrid structures in *in-vivo* human trials. Despite these advances, challenges remain in terms of production complexity and cost, as braiding and warp-knitting require specialized machinery and expertise, limiting large-scale manufacturing.

### 4.2 Skin

The skin is a highly complex organ composed of multiple layers, each with distinct structures and functions and a tensile strength of 2–14 N/mm^2^ (Table 1). The dermis provides mechanical strength and elasticity, while the underlying adipose tissue offers cushioning and insulation. There is a significant clinical need for the reconstruction of soft tissue defects resulting from tumor resection, deep burns or severe trauma. Textile scaffolds have shown to be effective in the creation of various skin tissues, including dermis and adipose layer [180,181].

#### 4.2.1 Dermis layer

For skin multiple successful tissues were fabricated using textile scaffolds. Ng and Hutmacher [144,182] have successfully fabricated a cohesive, mechanically stable dermal-like construct by combining PLGA weft-knits with fibroblast layers, featuring a tensile strength of 18.4 N/mm^2^ (Fig. 5 C). Chen et al. [183] also used a PLGA warp-knits and formed a hybrid scaffold with web-like collagen microsponges in the openings of the warp-knit. They demonstrated that the collagen microsponges in the hybrid fabric enhance the adherence and proliferation of fibroblasts and facilitate cell seeding with uniform cell distribution and thus the formation of homogeneous skin tissue (Fig. 5 D). Afrashi et al. [184] investigated the potential of egg white in combination with PCL and gelatin. The electrospun nanofiber mat is used as a scaffold for culturing fibroblasts. They showed that an increase in egg-white concentration decreases the hydrophilicity as well as the tensile strength and elongation at break (0 % egg white: approx. 3.9 MPa, 19 % elongation; 15 % egg white: approx. 1.2 MPa, 11 % elongation). Significantly enhanced cell growth compared to other concentrations was observed with a concentration of 10 % egg white. Dias et al. [185] discusses the use of electrospun materials in creating skin substitutes, highlighting the advantages and advancements in this field. They foresee several advances in this field, such as the development of combined and functional structures using different deposition strategies, the integration of different technologies (e.g. 3D printing) with electrospinning to obtain hybrid structures with tunable properties. For example, electrospun scaffolds with Young's modulus in the range of human skin of 2.9–82 N/mm^2^ are already reported. The same applies to the direct electrospinning of a solution of cells dissolved in polymers and the concept of *in-situ* electrospinning, which offers the possibility of depositing electrospun nanofibers directly on the wound without being limited by the size or depth of the wound.

#### 4.2.2 Adipose layer

Focusing on adipose tissue replacement, Shanti et al. [186,187] seeded hMSC onto an electrospun PLLA scaffold and successfully differentiated them into adipocytes, demonstrating the potential of this strategy for *in-vitro* engineering of adipose tissue grafts. Their scaffolds had a Young's modulus in the order of 8.5 N/mm^2^, which is in the known range of skin that typically contains adipose layers (Table 1). Schäfer et al. [188] used 3D warp-knitted PET spacer fabrics with a Young's modulus of 6.74 N/mm^2^ as an optimal three-dimensional scaffold for proliferation and differentiation of hMSC into the adipogenic line (Fig. 5 E). Compared to the knitted scaffold with a porosity of 43.4 %, the electrospun scaffold had a significantly higher porosity of over 90 %.

For planned reconstructive skin surgery with the possibility of using autologous cells, *in-vitro* approaches with knitted fabric of Ng and Hutmacher [144,182], Schäfer et al. [188] and Chen et al. [183] offer the advantages of high mechanical stability and voluminous graft material. However, for example Schäfer et al. use a non-degradable material (PET), making their scaffold a permanently implanted device. This can cause immunoreactions and inflammation. This limitation leaves room for further investigation of the structure but with suitable, degradable or even drug releasing materials [189]. Off-the-shelf scaffolds without cell integration are promising for immediate wound coverage and are particularly beneficial for preventing infections [190]. These could be e.g. thin, degradable electrospun nanofiber mats combined with hydrogels presented by Shanti et al. [186,187] or knitted spacer fabrics presented by Zhang et al. [190].

### 4.3 Cardiovascular system

The cardiovascular system includes the heart, blood vessels and associated structures. Due to cardiovascular conditions like valve dysfunction, heart damage and vascular diseases tissue engineered scaffolds are required. Textile scaffolds that can withstand dynamic mechanical forces have proven to be successful in this area [[191], [192], [193]].

#### 4.3.1 Blood vessels

Many research groups are working on the topic of blood vessels, and almost all textile methods have been used to produce replacements. For example, Hu et al. [194] used electrospinning to fabricate a small-diameter four-layer tubular scaffold consisting of three aligned layers and a random outer layer with tensile strength between 2.6 and 7 N/mm^2^, which is in the range of 0.03–4 N/mm^2^ of human blood vessels (Table 1). Fukunishi et al. [195] used the braiding method to produce a vascular graft consisting of rapidly degradable PGA. When implanted in a rat, they showed that the original scaffold was almost completely degraded after 6 months and a neoartery comparable to the native aorta with a tensile strength of 2.6 N/mm^2^ was formed. Wei et al. [196] investigated the structure as well as tensile and compressive properties of a tubular, braided scaffold of PEG-DE crosslinked bionic silk fibroin to evaluate its potential for use in small-diameter vascular grafts. Their grafts exhibited an axial tensile strength between 0.64 and 0.76 N/mm^2^ and a radial tensile strength between 12.1 and 21.6 N/mm^2^ (Fig. 5 F). Lin et al. [145] developed and tested braided, warp-knitted and weft-knitted vascular stents composed of twisted PVA yarns. The results of the *in-vivo* tests showed that PVA vascular stents had an intact structure after 28 days, indicating good biological properties. Finally, Liu et al. [197] developed a novel bifurcated stent graft with a seamless tubular structure using weaving technology, which avoids the leakage of conventional stent grafts. Depending on the fiber arrangement and treatment the tensile strength varies between 20 and 100 N/mm^2^. Rama et al. [198] developed warp-knitted biohybrid vascular grafts with an inner diameter of 1.5 mm containing PLGA fibers with superparamagnetic iron oxide nanoparticles or polyvinylidene fluoride scaffolds labeled with fluorinated TPU. These grafts enabled non-invasive MRI monitoring, e.g. of material degradation, demonstrating their potential for use in small human vessels such as coronary arteries.

#### 4.3.2 Heart valve

Another common application of textiles in regenerative medicine is artificial heart valves [147,199,200]. For example, Yousefi et al. [199] compare the mechanical and hemodynamic performance of woven polyester valves with biological valves *in-vitro*. Their test results show that textile valves trade their elasticity for higher mechanical strength compared to biological tissue, while the dynamic flexibility of the textile valve leaflets was similar to that of the biological valve leaflets. However, due to the greater porosity, regurgitation and slightly altered turbulence patterns were higher in textile valves than in biological valves. Weber et al. [147] used a warp-knit to design a tube-in-tube co-scaffold with improved mechanical properties for implantation in the aortic position. The design can be tailored to the patient's anatomy and has the potential to provide an autologous, living heart valve with growth capacity, physiological hemocompatibility and ease of implantation (Fig. 5 G). Ross et al. [200] combined a weft-knit and electrospun fibers to produce a heart valve and tested the opening of the valve and the bursting strength of the individual leaflets. The combination showed an improvement in the mechanical strength of the valve leaflets but also led to a reduction in their flexibility. Xu et al. [201] used melt electro writing of PCL in combination with GelMA and ChsMA coatings to fabricate an anisotropic heart valve. *In-vivo* tests in rats showed a significant reduction of immunoreaction and calcification of the coated scaffolds compared with PCL only scaffolds. They also showed that hemocompatibility and endothelization was supported by ChsMA.

#### 4.3.3 Heart muscle

Addressing the challenges of cardiac muscle, Boublik et al. [202] developed hybrid cardiac constructs consisting of heart cells and a biodegradable weft-knitted scaffold of Hyaluronan benzyl ester. Their scaffold exhibited mechanical properties suitable for *in-vitro* loading studies and *in-vivo* implantation (Fig. 5 H). Gupta et al. [203] studied the chemical and mechanical properties of electrospun polymer matrices (x%PEG-y%PCL-z%CPCL) for directed differentiation of stem cells towards a cardiomyogenic lineage. The polymers exhibited Young's moduli ranging from 7.5 to 23.21 MPa. Among the polymers evaluated, the polymer composition of 4 % PEG, 86 % PCL, and 10 % CPCL optimally facilitated the differentiation of ESCs into functional cardiomyocytes.

For TE of the cardiovascular system, the classical textiles (knits, weaves, braids) are used more frequently than electrospinning or MEW. Tubular scaffolds with a high kink resistance and pulsating mechanical deformation are necessary. Knitted materials offer both kink resistance and the ability to deform either as a leaflet of a heart valve, as a substitute for a blood vessel or as a scaffold for heart muscle cells. However, promising results of braided scaffolds were presented by Fukunishi et al. [195] as they achieved neo-artery growth within 6 months after implanting a PGA braided stent. The combination of a knitted structure with an electrospun coating used as a heart valve shows a reduced flexibility of the structure [200] pointing to the warp-knitted structure designed by Weber et al. [147] as a more promising option.

### 4.4 Respiratory system

For the respiratory system, especially the trachea, which comprises annular oriented cartilage rings, is tackled in tissue engineering with textiles. These tubular textile structures can again be produced either directly or by winding flat textiles to create structures with customized properties [[204], [205], [206], [207]].

**Fig. 6:**
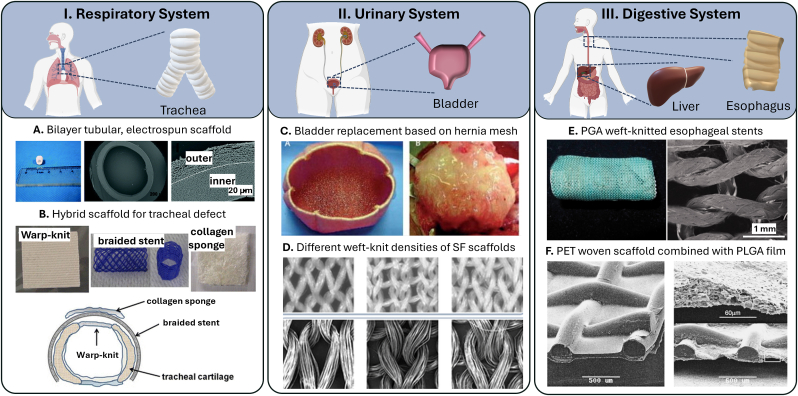
Overview of applications of textile-based scaffolds for tissue engineering in the respiratory, urinary, and digestive system. A. bilayer tubular scaffold, composed of electrospun collagen/P(LLA-CL) fibers [206], B. hybrid scaffold consisting of a PLGA knitted mesh and a collagen sponge to regenerate cartilage in a tracheal defect [207], C. collagen and a PGA mesh as an artificial bladder [213], D. different knit densities of weft-knitted silk fibroin scaffolds [215], E. knitted esophageal stents [221], F composite scaffold of macroscopically textiles and microscopically films [222].

For example, Wu et al. [206] demonstrated that their bilayer tubular scaffold, composed of electrospun collagen/P(LLA-CL) fibers, pre-processed with autologous tracheal cells and vascularization, is a promising alternative for tracheal tissue repair with an ultimate tensile strength of around 6.92 N/mm^2^ (Fig. 6 A). Tatekawa et al. [207] used a hybrid scaffold consisting of a PLGA warp-knit, a braided stent and a collagen sponge to regenerate cartilage in a tracheal defect. However, *in-vivo* experiments showed that the regenerated cartilage was not stiff enough to maintain the stiffness of the tracheal lumen (Young's modulus scaffold: 7.52 ± 1.60 × 10^−2^ N/mm^2^ and native trachea: 10.79 ± 1.49 × 10^−2^ N/mm^2^) (Fig. 6 B).

For tracheal tissue engineering, electrospun scaffolds such as those developed by Wu et al. [206] show potential for tracheal repair but fail to replicate the natural annular cartilage structure that may be critical for maintaining airway patency. Electrospinning produces randomly oriented fibers that lack the mechanical stiffness and organized architecture required for long-term functionality. In contrast, multi-technology approaches, such as the hybrid scaffold by Tatekawa et al. [207], offer a more structured design. However, even this approach failed to fully restore the native stiffness of the tracheal lumen *in-vivo*, highlighting the challenge of replicating the biomechanical properties of cartilage. Future studies should focus on refining hybrid scaffolds by integrating materials that better mimic the anisotropic structure and mechanical resilience of the native trachea.

### 4.5 Urinary system

The urinary system, particularly the bladder, often requires tissue engineering solutions due to high demand for bladder implants and repairs [[208], [209], [210], [211]]. Replacement of the bladder as a whole or in parts is required for a variety of clinical conditions, such as cancer [212]. One of the best-known examples of tissue engineering, which has also attracted public interest, is the bladder replacement by Anthony Atala and his team from the Wake Forest Institute for Regenerative Medicine in 2006 [213,214] (Fig. 6 C). Subsequently, other groups have conducted research in this direction using textile-based scaffolds for bladder reconstructions.

Khademolqorani et al. [215] investigated how different weft-knit densities (138, 182, and 245 stitches/cm^2^) affect the mechanical properties of silk fibroin scaffolds intended for tissue engineering in the urinary bladder. The study examined both uniaxial and multiaxial mechanical properties and found that cell culturing increased the strength and elongation of the scaffold over time. The weft-knit with 182 stitches/cm^2^ was found to closely resemble the porcine bladder, making it a particularly promising candidate for further tissue engineering studies in the bladder (Fig. 6 D). Besides weft-knitting, electrospinning has been applied for bladder regeneration. Feng et al. [216] report the fabrication of HA coated biomimetic nanofibrous scaffolds via coaxial electrospinning of PLCL and HA to modulate proliferation and migration of smooth muscle cells for bladder regeneration. Stankus et al. [217] combined an electrospun scaffold of biodegradable poly(ester-urethane)urea (PEUU) and a porcine ECM scaffold (urinary bladder matrix, UBM) to form a hybrid scaffold that was tested both *in-vitro* and *in-vivo* for its bioactive and physical properties. Their results showed that increasing amounts of PEUU led to a linear increase in tensile strength, while increasing amount of UBM led to higher adhesion and proliferation of smooth muscle cells *in-vitro* and increased mass loss *in-vitro.*

Despite Atala's early success, later research has focused on refining scaffold properties rather than advancing clinical translation. Studies such as those by Khademolqorani et al. [215] examined the effect of weft-knit density on mechanical properties. Similarly, Feng et al. [216] improved electrospun scaffolds with HA coatings to enhance cell proliferation, while Stankus et al. [217] combined PEUU with UBM to balance mechanical strength and bioactivity. Although these studies provide valuable insights into scaffold optimization, they represent a step back from whole organ engineering to more detailed material and mechanical investigations. Moving forward, the focus should return to clinically scalable solutions that bridge the gap between laboratory advancements and real-world applications.

### 4.6 Digestive system

The digestive system comprises several organs such as the esophagus, stomach, intestines, liver, and pancreas, each with distinct functions that necessitate tailored tissue engineering strategies. For instance, the esophagus requires scaffolds that can handle both mechanical stress and flexibility, while the liver demands structures that support complex cell functions and vascularization [[218], [219], [220], [221], [222], [223]].

#### 4.6.1 Esophagus

In the field of esophageal tissue engineering, Kuppan et al. [224] fabricated and investigated electrospun nanofiber mats as scaffolds. Their results suggest that aligned nanofibers of PHBV and PCL-gelatin could be promising scaffolds for esophageal tissue regeneration. Yekrang et al. [221] simulated different structures of weft-knitted esophageal stents using FE and mathematical models. After investigating them in axial and circumferential directions, they compared the mechanical behavior of these tubular structures. The results showed that the weft-knitted PGA stents with a Young's modulus of 0.61 N/mm^2^ can mimic the behavior of the esophagus in real applications (Fig. 6 E).

#### 4.6.2 Liver

Exploring innovative solutions for liver tissue engineering, Karamuk et al. [222] investigated a composite scaffold structure of macroscopically structured textiles and microscopically structured films (Fig. 6 F). PET woven textiles were used as a structuring scaffold and combined with a degradable, porous PLGA film and PVLA coating. An effect of the pore size on the aggregation behavior of hepatocytes was observed: with higher pore size, the rate of aggregation increased. It is suggested that textiles can be used as a fixing grid for cell aggregates. In a similar study, a PET woven fabric was coated with a degradable collagen-chitosan membrane. The scaffold was successfully used to grow HUVECs inside of the collagen-chitosan matrix and on its surface [223]. Furthermore, Ghahremanzadeh et al. [225] investigated the viability of cells on electrospun nanofiber mats using different galactosylation methods. Degradable scaffolds of PCL and chitosan were successfully functionalized by galactosylation. The hydrophilicity of the scaffold increased while the morphology and structure of the pores and the connection between the pores were maintained, leading to increased cell viability of HepG2 cells due to the available galactose on the scaffold surface.

For digestive system TE, scaffold requirements vary significantly depending on the organ. In esophageal reconstruction, electrospun scaffolds, as shown by Kuppan et al. [224], performed well, but the weft-knitted PGA stents, tested by Yekrang et al. [221], better mimicked the esophagus's mechanical behavior. Liver tissue engineering faces greater challenges due to complex cell organization and the need for vascular networks. Studies by Karamuk et al. [222] and Ghahremanzadeh et al. [225] showed that textile-based scaffolds, when combined with porous films, coatings, or biochemical functionalization improved hepatocyte aggregation and viability. However, these approaches still lack the vascular structures needed for long-term function. Future research could try using the fibrous structure of textiles to guide endothelial cell growth, helping to create functional vascularized engineered tissue [226].

### 4.7 Musculoskeletal system

The musculoskeletal system comprises various tissues that are important for movement and support, including muscles, tendons and ligaments, cartilage, and bone. Advances in tissue engineering are targeting these different components to improve repair and regeneration [[227], [228], [229], [230]]. In this section, applications of textile scaffolds in tissue engineering for the treatment of injuries and diseases of the entire musculoskeletal system are presented.

#### 4.7.1 Muscle

**Fig. 7:**
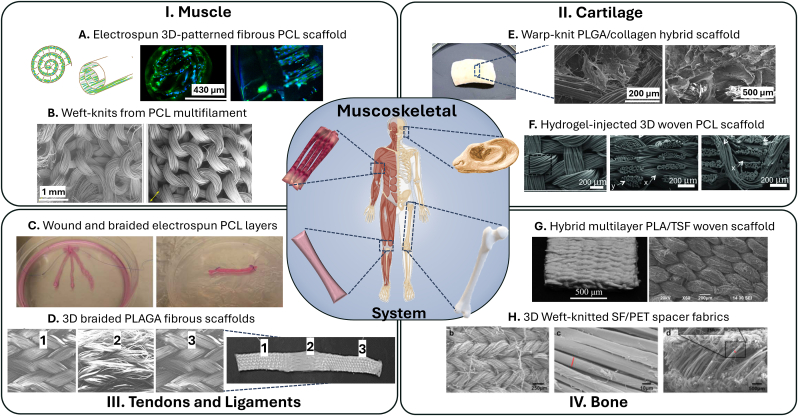
Overview of applications of textile-based scaffolds for musculoskeletal tissue engineering. A. Winded up electrospun 3D-patterned fibrous PCL scaffold [234], B. weft knitted PCL scaffold for regenerating facial skeletal muscle [235], C. braided electrospun PCL layers into a ligament construct [162], D. braided tissue-reinforced ligament scaffold [239,240], E. PLGA/collagen hybrid scaffold for cartilage regeneration [248], F. hydrogel-injected porous, three-dimensionally woven PCL scaffold mimicking cartilage [173], G hybrid multilayer electrospun and woven PLA/TSF scaffolds [158], H. 3D spacer fabrics made of weft-knitted silk fibroin yarns (SF) with a PET spacer layer for bone regeneration [255].

Since muscles are composed of organized and aligned myofibrils and myotubes, muscle scaffolds should ideally mimic this micro-/nanotopography of natural muscle and have suitable mechanical properties (e.g. tensile strength 0.01–0.02 N/mm^2^ and Young's Modulus 0.01–0.45 N/mm^2^, Table 1) [231,232]. Thangadurai et al. [233] have reviewed novel strategies for the integration of electrospinning and bioprinting to produce skeletal muscle tissue constructs. They see the combination of electrospinning and bioprinting as a major key to success in the clinical implementation of bioengineered skeletal muscle constructs for regenerative medicine applications. For example, they reported Zhang et al. [234], who developed a hybrid electrospun and MEW patterned PCL scaffold with a Young's modulus of 0.78 N/mm^2^, which is in the range of natural tissue (Fig. 7 A). Deshpande et al. [235] developed weft-knitted scaffolds from poly(ε-caprolactone) multifilament yarns, designed with an auxetic structure that has a negative Poisson's ratio, for regenerating facial skeletal muscle. These resorbable scaffolds, with over 90 % porosity, were evaluated for their physical properties, mechanical performance, and biocompatibility using neonatal human dermal fibroblasts. The scaffolds exhibited superior bursting strength (0.9 N/mm^2^) and biaxial elongation (300 %) compared to native skeletal muscle tissue (1.075 N/mm^2^, 65 %), ensuring adequate mechanical support post-implantation (Fig. 7 B).

For muscle regeneration, scaffolds should mimic the alignment and size of natural muscle fibers. Hybrid methods combining electrospinning with bioprinting [233] or MEW [234] could match muscle stiffness and muscle fiber size (20–100 μm). Deshpande et al. [235] improved strength and flexibility with weft-knitted auxetic scaffolds. However, most textile fibers still exceed the stiffness and size of myofibrils (1–2 μm), limiting biomimicry. Despite these advancements, clinical translation remains challenging, requiring further *in-vitro* and *in-vivo* validation.

#### 4.7.2 Tendons and ligaments

Ligament or tendon ruptures are common injuries. Current treatment methods can lead to an adequate reconstruction of the damaged ligament, but have significant disadvantages, mainly related to the reduced availability of tissue and the pain associated with tissue removal [[236], [237], [238]]. Vaquette et al. [162] cultivated mesenchymal stem cells on electrospun PCL layers, wound them up and braided them by hand into a ligament construct. Within the construct and during tissue integration *in-vivo*, promising results were obtained in terms of homogeneity of cell distribution. However, with a tensile strength of around 0.02 N/mm, the scaffold remained significantly below that of native tissue (Fig. 7 C). Cooper et al. [239,240] used 3D braiding technology to produce a tissue-reinforced ligament scaffold that has the mechanical properties such as a tensile strength of 100–400 N/mm^2^ of the normal anterior cruciate ligament and a porosity of 55–67 % for tissue ingrowth (Fig. 7 D). Emonts et al. [241] investigated PCL braids as degradable cruciate ligament repair. High draw ratios during the melt spinning process as well as a multilayer braid design yielded PCL braids with maximum tensile load of 4750 N, exceeding the required maximal tensile force by a factor of two. Sahoo et al. [242] compared weft-knitted and hand woven scaffolds. Their weft-knitted scaffolds showed better cell adhesion and proliferation than their woven scaffolds. In a follow-up study [243], they produced a biohybrid scaffold of electrospun PLGA fibers on a weft-knitted scaffold of degummed silk. These constructs closely mimicked the natural ECM nanoarchitecture and facilitated cell seeding. They were able to stimulate initial proliferation and subsequent tenogenic differentiation, which increased collagen production and led to stronger constructs with a stiffness of 5–7 N/mm.

Tendon and ligament tissue engineering requires scaffolds that withstand high mechanical loads while promoting cell integration. Vaquette et al. [162] used electrospun PCL layers to create ligament constructs, but their tensile strength remained below that of native tissue. In contrast, Cooper et al. [239,240] achieved native-level properties using 3D-braided tissue-reinforced scaffolds, and Emonts et al. [241] exceeded necessary mechanical strength with PCL braids. While these approaches advance biomimicry, long-term performance and degradation behavior *in-vivo* require further study. With its ability to provide high tensile strength in the axial direction, braiding may be the most suitable technology for tendon and ligament scaffolds.

#### 4.7.3 Cartilage

Cartilage tissue engineering is of great interest due to the limited self-healing capacity of cartilage and the high demand for effective treatments for injuries and degenerative diseases such as osteoarthritis. Unlike other tissues, cartilage lacks blood vessels, which makes repair and regeneration particularly difficult [[244], [245], [246]]. Focusing on this, Ahn et al. [247] designed and fabricated a multilayered braided 3D scaffold with axial filaments that mimics the anisotropic and gradient mechanical properties of natural articular cartilage, such as a compressive modulus of 0.58 N/mm^2^ and a Young's modulus between 1.4 and 37.9 N/mm^2^ (human cartilage: 1.16–7.75 N/mm^2^, 0.7–15.3 N/mm^2^, Table 1). The scaffold was designed based on numerical analysis, and comparison of the mechanical properties of the scaffold with the predicted results showed good agreement, confirming the validity of this prediction approach. Dai et al. [248] developed a PLGA/collagen hybrid scaffold combining the advantages of natural type I collagen and synthetic polymer warp-knit and used this scaffold for cartilage regeneration with controllable shape. The samples showed spatially uniform cell distribution, natural chondrocyte morphology and deposition of a rich cartilaginous extracellular matrix. The Young's modulus of the engineered cartilage was around 18.73 N/mm^2^ and compared to native articular cartilage, the mechanical strength reached 71.43 % of the stiffness (Fig. 7 E). Liao et al. [173] introduced the concept of injecting a mechanically robust network hydrogel of alginate and polyacrylamide into a porous, three-dimensionally woven PCL scaffold to create a construct with tunable mechanical and tribological properties that mimics the properties of native cartilage, such as a Young's modulus between 0.1 and 1.2 N/mm^2^ and offers a potential acellular or cell-based replacement for cartilage repair (Fig. 7 F).

Cartilage tissue engineering remains challenging due to its avascular nature and limited self-healing capacity. Ahn et al. [247] developed a multilayered braided scaffold that mimicked cartilage anisotropy, while Dai et al. [248] combined PLGA and collagen in a warp-knit scaffold to enhance chondrocyte distribution and extracellular matrix formation. Liao et al. [173] introduced a hydrogel-infused woven PCL scaffold, offering tunable mechanical properties. While these approaches improve biomimicry and mechanical performance, ensuring long-term durability and integration into native tissue remains an obstacle. Due to its avascular nature, acellular solutions may also be promising, potentially simplifying clinical translation.

#### 4.7.4 Bone

Bone tissue engineering addresses the significant need for effective treatments for fractures, bone defects and degenerative diseases such as osteoporosis. Due to its weight-bearing function and complex structure, bone requires scaffolds that can replicate its mechanical strength (tensile strength cancellous bone 1–5 N/mm^2^, cortical bone 60–193 N/mm^2^, Table 1), promote osteogenesis and integrate seamlessly with existing tissue [[249], [250], [251], [252]]. To treat bone defects of critical size, Xue et al. [253] fabricated 3D woven fabrics with magnesium wires that have a controlled chemical composition and microstructure. After weaving, they coated the wires with polylactic acid to increase their strength and corrosion resistance. They then characterized their mechanical properties, corrosion behavior and cell compatibility *in-vitro* and evaluated their corrosion behavior and fabric response *in-vivo*. The coated scaffolds had a Young's modulus of 1021.8 and 1259.1 N/mm^2^ making them much stiffer than natural bone. Shao et al. [158] used electrospinning and conventional textile weaving to produce PLA/TSF scaffolds with a Young's modulus of 417.65 N/mm^2^ and a tensile strength of 180.36 N/mm^2^ that mimic the hierarchical architecture of the collagen fibril matrix in natural lamellar bone. *In-vitro* tests showed that the woven scaffold not only promoted MSC adhesion and proliferation, but also increased osteogenesis, alkaline phosphatase activity and mineralization compared to a control group (Fig. 7 G). Rentsch et al. [254] demonstrated that their embroided mesh and surface-coated scaffolds serve as a temporary matrix for cell migration, proliferation, differentiation, and vascularization in bone tissue engineering. The embroided scaffolds were stacked to create a 3D implant with sufficient porosity and fully interconnecting pores. Seeded hMSC were able to survive 6 weeks of subcutaneous implantation and differentiated in an osteogenic direction. Ribeiro et al. [255] compared 3D spacer fabrics made of weft-knitted silk fibroin yarns (SF) with a PET spacer layer with warp-knitted PET-only structures for bone regeneration in the craniofacial complex (Fig. 7 H). Here, SF-PET weft-knit spacer fabrics showed higher mechanical properties and promoted osteogenic differentiation of human adipose-derived stem cells under osteogenic culture conditions. Compared with the compressive modulus of human bone (2–230 N/mm^2^, Table 1), it was similarly low for both structures (0.041 N/mm^2^ for SF-PET, 0.039 N/mm^2^ for PET). In addition, SF-PET scaffolds showed an improved angiogenic response in a chick chorioallantoic membrane assay and supported tissue ingrowth with blood vessel infiltration in a mouse model, suggesting their potential as tissue engineering scaffolds for craniofacial bone regeneration.

Although acellular bone implants exist, tissue engineering remains crucial for addressing large defects and improving biological integration. 3D-woven magnesium scaffolds have shown improved strength but can be too stiff compared to natural bone [253]. Hybrid scaffolds of electrospun and woven PLA/TSF showed the ability to enhance osteogenesis by trying to replicate the hierarchical structure of bone [158]. Rentsch et al. [254] stacked multiple 2D embroidered scaffolds for improved vascularization, while Ribeiro et al. [255] showed that their 3D silk fibroin-PET spacer fabrics promoted osteogenic differentiation and angiogenesis. While each approach addresses a specific aspect of bone TE (e.g. improve mechanical or biological performance), none of the reviewed fully capture the combined requirements for a functional, long-lasting TE bone.

## 5 Summary and outlook

The combination of textile engineering and cell culture methods provides wide possibilities for tissue engineering applications. Most textile-based scaffolds aim to mimic the mechanical properties of the native tissue. However, the extremes of soft and hard tissues such as muscle or bone remain challenging (Fig. 4). This is due to the highly complex interaction between material, fiber properties, textile technology and textile design, which leads to this wide range of possible properties, making the selection process when designing a scaffold a challenge.

Fiber formation technologies are selected based on the polymer material and desired properties. Melt spinning is ideal for thermoplastics like polyamide and polylactide, offering a cost-effective and solvent-free process. Solution spinning suits polymers such as collagen and silk fibroin but may leave solvent residues. It enables the creation of highly porous fibers. Electro- and microfluidic spinning are specialized methods for producing nanofibers. Independent of the technology chosen, the raw material, fiber and textile should be tested for biocompatibility in *in-vitro* cytotoxicity tests.

Each textile technology offers distinct advantages for tissue engineering and can be tailored to meet specific mechanical and biological requirements (Fig. 4). Weaving may provide high mechanical strength and controlled porosity, making it suitable for creating durable and precisely structured scaffolds. Braiding offers flexibility and a high strength-to-weight ratio. All yarns used in a braid are orientated mainly in the longitudinal direction, which leads to high tensile strength. Knitting has high structural elasticity, which can be beneficial for dynamic tissues that undergo frequent deformation. In general, porosity, pore size and mechanical properties of warp-knits are more customizable than those of weft-knits and can be designed in larger ranges. However, the pore size of weft-knits and elastic warp-knits largely depends on the applied stretch of the textile.

While textile scaffolds can be optimized for various applications, the selection of a specific technology depends on the target tissue, required mechanical properties, and available fabrication methods. Hereby, some technologies may be more suitable for some applications than others (Table 3). Braided scaffolds, with their longitudinal orientation of fibers, may be best suited for axially loaded tissues such as tendons and ligaments. In addition, braiding enables the production of stable hollow lumens that can be used in venous scaffolds as well as circular weft-knitted textiles. Flat textiles produced by weaving, weft- or warp-knitting can be chosen depending on the availability of the technology, with warp-knitting being recommended for the greatest flexibility in structure and design. Spacer fabrics extend the potential of 2D textiles to 3D scaffolds [127,188,255] and are particularly promising for applications such as skin grafts or multilayer structures. For researchers without direct access to textile manufacturing, electrospinning remains the most accessible method, as it requires fewer pre-processing steps such as yarn fabrication. It can produce nanofibers with a high surface area, closely mimicking the extracellular matrix to enhance cell attachment and growth. However, its structural limitations must be considered.

**Table 3:** Summary of key advantages, limitations and suitable applications for reviewed textile technologies.

Textile technology	Key advantages	Limitations	Suitable application
**Weaving** [118,127,133]	• High mechanical strength • Controlled small porosity • Possible sealing	• Loose filaments when cut • Elasticity	Vessels and cartilage
**Braiding** [137,140,141]	• High mechanical strength in longitudinal direction • Possibility for open lumen	• Variations in structure • Width of flat braid	Tendon and ligaments, conduits and vessels
**Weft-Knitting** [134,135]	• High structural elasticity • Adjustable porosity • Flat and round textiles possible	• Non-sealing • Mechanical strength • Complexity of machining	Skin, heart valves, soft tissue
**Warp-Knitting** [[134], [135], [136]]	• Adjustable structural elasticity • Adjustable porosity and mechanical properties • Variety in design	• Non-sealing • Complexity of machining	Skin, heart valves, soft tissue
**Electrospinning** [125,126]	• (Nano-)Fiber and textile formation in one step • High surface area for cell attachment • Combinable with other textiles	• Weak structural integrity • Limited height and cell infiltration • Fibers only random or aligned	Barriers in conduits or skin, combination with other scaffolds
**Melt Electro Writing** [132,166]	• (Nano-)Fiber and textile formation in one step • Freely designable structures	• Limited scalability • Max height 10 mm	Cartilage, skin, combination with other scaffolds

Regardless of the technology used, textile structures (e.g. stitches) do not naturally exist in biological tissues, and their impact on cell behavior, particularly for alignment-dependent applications, require careful evaluation. Achieving nonlinear mechanical behavior and radial compliance, such as in vascular grafts, necessitates fine-tuning. Despite numerous *in-vitro* and preclinical successes, human trials remain rare, raising concerns about whether textile-based scaffolds are advancing toward clinical application or merely undergoing incremental refinements without real-world translation. *In-vivo* studies, such as those by Politikou et al. [179], are critical to bridging this gap.

Future research needs to focus on scalable and clinically feasible solutions rather than isolated material optimizations to ensure that textile-based scaffolds move beyond laboratory development into viable medical applications. While textile production is not the limiting factor here, the manual processing of most textile scaffolds in TE is. For this reason, the use of large textile machines for small-scale production can often be excessive and labor intensive. Looking ahead, advances in tissue engineering could benefit from techniques such as 3D screen printing [256,257], which could enable upscaled applications with textile scaffolds. For personalized applications and as long as these techniques are not yet established, 3D printing and melt electro-writing offer promising solutions as they enable the production of intricate, customized textile scaffolds [132,258]. Besides technical scalability, if the size of the engineered tissue exceeds the diffusion limit for nutrients, some form of vascularization is required, which poses further challenges.

Furthermore, advancements in textile technology open possibilities for multifunctional bioreactors, wearable devices, and biosensors. Inspired by the concept of self-pumping spacer fabrics, textiles could be engineered as microfluidic bioreactors, enabling dynamic cell culture environments [259]. Additionally, conductive fibers have the potential to facilitate electrostimulation [260,261], which could be harnessed for regeneration of nerves [262] and muscles [234]. By adapting fiber shapes and improving conductivity, textiles could enable health monitoring systems powered by energy generated by the human body, such as motion, pressure, and heat, serving as platforms for advanced biosensors [[263], [264], [265]].

The future of tissue engineering is promising and is driven by the interdisciplinary collaboration of textile technology, materials science and biomedical research. Both research into implantable textiles, whose patterning and properties are tailored to body tissue, and research into novel solutions for tissue regeneration have the potential to advance the field of TE. By innovative technologies such as 3D bioprinting and advanced biosensors, the integration of textiles into tissue engineering is leading to a new era of personalized and regenerative medicine, offering the prospect of better medical outcomes and a higher quality of life.

## CRediT authorship contribution statement

**S. Scholpp:** Writing – review & editing, Writing – original draft, Visualization, Methodology, Conceptualization. **L.A. Hoffmann:** Writing – review & editing, Visualization, Methodology. **E. Schätzlein:** Writing – review & editing, Supervision. **T. Gries:** Writing – review & editing, Supervision, Funding acquisition. **C. Emonts:** Writing – review & editing, Supervision. **A. Blaeser:** Writing – review & editing, Visualization, Supervision, Funding acquisition, Conceptualization.

## Declaration of competing interest

The authors declare that they have no known competing financial interests or personal relationships that could have appeared to influence the work reported in this paper.

## Data Availability

Data will be made available on request.

## Footnotes

2 Manufacturer information: https://www.lindauerdornier.com/de/webmaschinen/luftwebmaschine-a2/.

3 Manufacturer information: https://www.mayercie.com/en/braiding-machines/machine-selection-mbt/.

4 Manufacturer information: https://www.mayercie.com/en/circular-knitting-machines/machine-selection/.

5 Manufacturer information: https://www.elmarco.com/product-lines/infinity.

## References

[1] Brockmann I. (2018). Skin-derived stem cells for wound treatment using cultured epidermal autografts: clinical applications and challenges. Stem Cells Int..

[2] Ren J. (Feb. 2022). Reconstruction of the trachea and carina: surgical reconstruction, autologous tissue transplantation, allograft transplantation, and bioengineering. Thorac. Cancer.

[3] Habka D., Mann D., Landes R., Soto-Gutierrez A. (Jul. 2015). Future economics of liver transplantation: a 20-year cost modeling forecast and the prospect of bioengineering autologous liver grafts. PLoS One.

[4] Shen Y. (Jun. 2022). Cancer risk and mutational patterns following organ transplantation. Front. Cell Dev. Biol..

[5] Shandalov Y. (Apr. 2014). An engineered muscle flap for reconstruction of large soft tissue defects. Proc. Natl. Acad. Sci. U. S. A..

[6] Ferreira L.M., Ferreira L.R.K. (2004). Experimental model for composite tissue allotransplantations. Acta Cir. Bras..

[7] Sosa E. (Dec. 2018). Differentiation of primate primordial germ cell-like cells following transplantation into the adult gonadal niche. Nat. Commun..

[8] Health Resources and Services Administration, “Organ Donation Statistics | Organdonor.gov,” Organdonor.Gov. Accessed: Dec. 30, 2023. [Online]. Available: https://www.organdonor.gov/learn/organ-donation-statistics.

[9] Edgar L. (Jun. 2020). Regenerative medicine, organ bioengineering and transplantation. Br. J. Surg..

[10] Fisher J.P., Mikos A.G., Bronzino J.D. (2007). Tissue engineering. Tissue Eng..

[11] Heidary Rouchi A., Mahdavi-Mazdeh M. (2015). Regenerative medicine in organ and tissue transplantation: shortly and practically achievable?. Int. J. Organ Transplant. Med..

[12] Ikada Y. (Oct. 2006). Challenges in tissue engineering. J. R. Soc. Interface.

[13] Inzana J.A. (Apr. 2014). 3D printing of composite calcium phosphate and collagen scaffolds for bone regeneration. Biomaterials.

[14] Kang H.W., Lee S.J., Ko I.K., Kengla C., Yoo J.J., Atala A. (Feb. 2016). A 3D bioprinting system to produce human-scale tissue constructs with structural integrity. Nat. Biotechnol..

[15] Keriquel V. (Dec. 2017). In situ printing of mesenchymal stromal cells, by laser-assisted bioprinting, for in vivo bone regeneration applications. Sci. Rep..

[16] Lemaire S.A., Weldon S.A., Coselli J.S. (2013). Total aortic arch replacement: current approach using the trifurcated graft technique. Ann. Cardiothorac. Surg..

[17] Amirghofran A.A., Karimi A., Emaminia A., Sharifkazemi M.B., Salaminia S. (2011). Brucellosis relapse causing prosthetic valve endocarditis and aortic root infective pseudoaneurysm. Ann. Thorac. Surg..

[18] Li C., Wang F., Douglas G., Zhang Z., Guidoin R., Wang L. (May 2017). Comprehensive mechanical characterization of PLA fabric combined with PCL to form a composite structure vascular graft. J. Mech. Behav. Biomed. Mater..

[19] Petrulyte S., Petrulis D. (2011). Handbook of Medical Textiles.

[20] Lanza R.P., Langer R., Vacanti J. (2000).

[21] Doser M., Planck H. (Jan. 2011). Textiles for implants and regenerative medicine. Handb. Med. Text..

[22] Sengupta D., Waldman S.D., Li S. (Jul. 2014). From in vitro to in situ tissue engineering. Ann. Biomed. Eng..

[23] Godbey W.T., Atala A. (Jun. 2002). In vitro systems for tissue engineering. Ann. N. Y. Acad. Sci..

[24] Li S., Sengupta D., Chien S. (Jan. 2014). Vascular tissue engineering: from in vitro to in situ. Wiley Interdiscip. Rev. Syst. Biol. Med..

[25] Ko I.K., Lee S.J., Atala A., Yoo J.J. (Nov. 2013). In situ tissue regeneration through host stem cell recruitment. Exp. Mol. Med..

[26] De Kort B.J. (Nov. 2021). Inflammatory and regenerative processes in bioresorbable synthetic pulmonary valves up to two years in sheep–Spatiotemporal insights augmented by Raman microspectroscopy. Acta Biomater..

[27] Emerson A.E., Mccall A.B., Brady S.R., Slaby E.M., Weaver J.D. (Sep. 2022). Hydrogel injection molding to generate complex cell encapsulation geometries. ACS Biomater. Sci. Eng..

[28] Tosoratti E., Bonato A., Kessel B., Weber P., Zenobi-Wong M. (May 2023). Shape-defining alginate shells as semi-permeable culture chambers for soft cell-laden hydrogels. Biofabrication.

[29] Li Y., Ma T., Kniss D.A., Lasky L.C., Yang S.T. (Jan. 2001). Effects of filtration seeding on cell density, spatial distribution, and proliferation in nonwoven fibrous matrices. Biotechnol. Prog..

[30] Zhang Y. (Sep. 2019). Polymer fiber scaffolds for bone and cartilage tissue engineering. Adv. Funct. Mater..

[31] Zhao P., Gu H., Mi H., Rao C., Fu J., sheng Turng L. (Dec. 2017). Fabrication of scaffolds in tissue engineering: a review. Front. Mech. Eng..

[32] Murphy S.V., Atala A. (2014). 3D bioprinting of tissues and organs. Nat. Biotechnol..

[33] Teßmar J., Brandl F., Göpferich A. (Jul. 2009). Hydrogels for tissue engineering. Fundam. Tissue Eng. Regen. Med..

[34] Blaeser A., Duarte Campos D.F., Fischer H. (Jun. 2017). 3D bioprinting of cell-laden hydrogels for advanced tissue engineering. Curr. Opin. Biomed. Eng..

[35] Tomasina C., Bodet T., Mota C., Moroni L., Camarero-Espinosa S. (Aug. 2019). Bioprinting vasculature: materials, cells and emergent techniques. Materials.

[36] Huang G. (Mar. 2024). Applications, advancements, and challenges of 3D bioprinting in organ transplantation. Biomater. Sci..

[37] Kolesky D.B., Homan K.A., Skylar-Scott M.A., Lewis J.A. (Mar. 2016). Three-dimensional bioprinting of thick vascularized tissues. Proc. Natl. Acad. Sci. U. S. A..

[38] Janmohammadi M., Nourbakhsh M.S. (May 2020). Recent advances on 3D printing in hard and soft tissue engineering. Int. J. Polym. Mater. Polym. Biomater..

[39] Stratton S., Manoukian O.S., Patel R., Wentworth A., Rudraiah S., Kumbar S.G. (Jun. 2018). Polymeric 3D printed structures for soft-tissue engineering. J. Appl. Polym. Sci..

[40] Dhandayuthapani B., Yoshida Y., Maekawa T., Kumar D.S. (2011). Polymeric scaffolds in tissue engineering application: a review. Int. J. Polym. Sci..

[41] Zhang Y.S., Khademhosseini A. (May 2017). Advances in engineering hydrogels. Science.

[42] Nie K. (Aug. 2020). Enzyme-crosslinked electrospun fibrous gelatin hydrogel for potential soft tissue engineering. Polymers.

[43] Park S.-H., Jung C.S., Min B.-H. (Dec. 2016). Advances in three-dimensional bioprinting for hard tissue engineering. Tissue Eng. Regen. Med..

[44] Olaru M., Sachelarie L., Calin G. (May 2021). Hard dental tissues regeneration—approaches and challenges. Materials.

[45] Baino F., Fiume E., Miola M., Verné E. (Jul. 2018). Bioactive sol-gel glasses: processing, properties, and applications. Int. J. Appl. Ceram. Technol..

[46] Cichos S. (May 2023). A new 3D-printed polylactic acid-bioglass composite for bone tissue engineering induces angiogenesis in vitro and in ovo. Int. J. Bioprinting.

[47] Zhang F., Li Z., Xu M., Wang S., Li N., Yang J. (Jul. 2022). A review of 3D printed porous ceramics. J. Eur. Ceram. Soc..

[48] Turnbull G. (Sep. 2018). 3D bioactive composite scaffolds for bone tissue engineering. Bioact. Mater..

[49] Zuo W., Yu L., Lin J., Yang Y., Fei Q. (Aug. 2021). Properties improvement of titanium alloys scaffolds in bone tissue engineering: a literature review. Ann. Transl. Med..

[50] Lichte P., Pape H.C., Pufe T., Kobbe P., Fischer H. (Jun. 2011). Scaffolds for bone healing: concepts, materials and evidence. Injury.

[51] Vedadghavami A. (Oct. 2017). Manufacturing of hydrogel biomaterials with controlled mechanical properties for tissue engineering applications. Acta Biomater..

[52] Norahan M.H., Pedroza-González S.C., Sánchez-Salazar M.G., Álvarez M.M., Trujillo de Santiago G. (Jun. 2023). Structural and biological engineering of 3D hydrogels for wound healing. Bioact. Mater..

[53] Hill E., Boontheekul T., Mooney D.J. (Feb. 2006). Regulating activation of transplanted cells controls tissue regeneration. Proc. Natl. Acad. Sci. U. S. A..

[54] Lesman A., Koffler J., Atlas R., Blinder Y.J., Kam Z., Levenberg S. (Nov. 2011). Engineering vessel-like networks within multicellular fibrin-based constructs. Biomaterials.

[55] Goldshmid R., Simaan‐Yameen H., Ifergan L., Loebel C., Burdick J.A., Seliktar D. (Sep. 2023). Modulus‐dependent effects on neurogenic, myogenic, and chondrogenic differentiation of human mesenchymal stem cells in three‐dimensional hydrogel cultures. J. Biomed. Mater. Res., Part A.

[56] Bilici Ç., Tatar A.G., Şentürk E., Dikyol C., Koç B. (Feb. 2022). Bisulfite-initiated crosslinking of gelatin methacryloyl hydrogels for embedded 3D bioprinting. Biofabrication.

[57] Levental I., Georges P.C., Janmey P.A. (Feb. 2007). Soft biological materials and their impact on cell function. Soft Matter.

[58] Carey S.P., Kraning-Rush C.M., Williams R.M., Reinhart-King C.A. (Jun. 2012). Biophysical control of invasive tumor cell behavior by extracellular matrix microarchitecture. Biomaterials.

[59] Anerillas L.O., Kingham P.J., Lammi M.J., Wiberg M., Kelk P. (Dec. 2021). Three-dimensional osteogenic differentiation of bone marrow mesenchymal stem cells promotes matrix metallopeptidase 13 (Mmp13) expression in type i collagen hydrogels. Int. J. Mol. Sci..

[60] Plou J., Juste-Lanas Y., Olivares V., del Amo C., Borau C., García-Aznar J.M. (Aug. 2018). From individual to collective 3D cancer dissemination: roles of collagen concentration and TGF-β. Sci. Rep..

[61] Sarrigiannidis S.O., Rey J.M., Dobre O., González-García C., Dalby M.J., Salmeron-Sanchez M. (Mar. 2021). A tough act to follow: collagen hydrogel modifications to improve mechanical and growth factor loading capabilities. Mater. Today Bio.

[62] Sánchez-Téllez D., Téllez-Jurado L., Rodríguez-Lorenzo L. (Dec. 2017). Hydrogels for cartilage regeneration, from polysaccharides to hybrids. Polymers.

[63] Niemczyk-Soczynska B., Zaszczyńska A., Zabielski K., Sajkiewicz P. (Nov. 2021). Hydrogel, electrospun and composite materials for bone/cartilage and neural tissue engineering. Materials.

[64] Wu S., Duan B., Qin X., Butcher J.T. (Mar. 2017). Living nano-micro fibrous woven fabric/hydrogel composite scaffolds for heart valve engineering. Acta Biomater..

[65] Engler A.J., Sen S., Sweeney H.L., Discher D.E. (Aug. 2006). Matrix elasticity directs stem cell lineage specification. Cell.

[66] Burg K.J.L., Porter S., Kellam J.F. (Dec. 2000). Biomaterial developments for bone tissue engineering. Biomaterials.

[67] Murphy C.M., Haugh M.G., O'Brien F.J. (Jan. 2010). The effect of mean pore size on cell attachment, proliferation and migration in collagen-glycosaminoglycan scaffolds for bone tissue engineering. Biomaterials.

[68] Artel A., Mehdizadeh H., Chiu Y.-C., Brey E.M., Cinar A. (Sep. 2011). An agent-based model for the investigation of neovascularization within porous scaffolds. Tissue Eng. Part A.

[69] Lien S.M., Ko L.Y., Huang T.J. (Feb. 2009). Effect of pore size on ECM secretion and cell growth in gelatin scaffold for articular cartilage tissue engineering. Acta Biomater..

[70] O'Brien F.J. (Mar. 2011). Biomaterials & scaffolds for tissue engineering. Mater. Today.

[71] Jia Z., Xu X., Zhu D., Zheng Y. (Apr. 2023). Design, printing, and engineering of regenerative biomaterials for personalized bone healthcare. Prog. Mater. Sci..

[72] Jiao Y. (Jun. 2020). Construction and application of textile-based tissue engineering scaffolds: a review. Biomater. Sci..

[73] Liu Y., Ji Y., Ghosh K., Clark R.A.F., Huang L., Rafailovich M.H. (Sep. 2009). Effects of fiber orientation and diameter on the behavior of human dermal fibroblasts on electrospun PMMA scaffolds. J. Biomed. Mater. Res., Part A.

[74] Badami A.S., Kreke M.R., Thompson M.S., Riffle J.S., Goldstein A.S. (Feb. 2006). Effect of fiber diameter on spreading, proliferation, and differentiation of osteoblastic cells on electrospun poly(lactic acid) substrates. Biomaterials.

[75] Beachley V., Wen X. (Jul. 2010). Polymer nanofibrous structures: fabrication, biofunctionalization, and cell interactions. Prog. Polym. Sci..

[76] Kreger S.T. (Aug. 2010). Polymerization and matrix physical properties as important design considerations for soluble collagen formulations. Biopolymers.

[77] Achilli M., Mantovani D. (Dec. 2010). Tailoring mechanical properties of collagen-based scaffolds for vascular tissue engineering: the effects of pH, temperature and ionic strength on gelation. Polymers.

[78] Kim J., Lee S., Gu C.-Y., Kim T.-S., Kong H., Jang D. (Nov. 2023). Mechanical characterization of collagen hydrogels by quasistatic uniaxial tensile experiments. Adv. Eng. Mater..

[79] Kramschuster A., Turng L.S. (Feb. 2010). An injection molding process for manufacturing highly porous and interconnected biodegradable polymer matrices for use as tissue engineering scaffolds. J. Biomed. Mater. Res. Part B Appl. Biomater..

[80] Damien C.J., Parsons J.R. (Sep. 1991). Bone graft and bone graft substitutes: a review of current technology and applications. J. Appl. Biomater..

[81] Nga N.K., Huyen T.T.T., Dung T.N. (Oct. 2023). Solvent casting-particulate leaching synthesis of a nano-SiO2/chitosan composite scaffold for potential use in bone tissue engineering. Vietnam J. Chem..

[82] Bruyère Garnier K., Dumas R., Rumelhart C., Arlot M.E. (Nov. 1999). Mechanical characterization in shear of human femoral cancellous bone: torsion and shear tests. Med. Eng. Phys..

[83] Cetinel O., Esen Z., Yildirim B. (Mar. 2019). Fabrication, morphology analysis, and mechanical properties of ti foams manufactured using the space holder method for bone substitute materials. Metals.

[84] Guimarães C.F., Gasperini L., Marques A.P., Reis R.L. (Feb. 2020). The stiffness of living tissues and its implications for tissue engineering. Nat. Rev. Mater..

[85] Morgan E.F., Unnikrisnan G.U., Hussein A.I. (2018). Bone mechanical properties in healthy and diseased states. Annu. Rev. Biomed. Eng..

[86] Meriç G., Erkmen E., Kurt A., Eser A., Çelik G. (Dec. 2010). Biomechanical evaluation of a fiber-reinforced composite prosthesis supported by implants with and without a microthread collar design. J. Dent. Sci..

[87] Dong X.N., Luo Q., Wang X. (Mar. 2013). Progressive post-yield behavior of human cortical bone in shear. Bone.

[88] Paul S. (Dec. 2023). Photo-cross-linkable, injectable, and highly adhesive GelMA-glycol chitosan hydrogels for cartilage repair. Adv. Healthc. Mater..

[89] Griffin D.J. (Jul. 2015). Mechanical characterization of matrix-induced autologous chondrocyte implantation (MACI®) grafts in an equine model at 53 weeks. J. Biomech..

[90] Jeon J.E., Schrobback K., Hutmacher D.W., Klein T.J. (Aug. 2012). Dynamic compression improves biosynthesis of human zonal chondrocytes from osteoarthritis patients. Osteoarthr. Cartil..

[91] Singh G., Chanda A. (Oct. 2021). Mechanical properties of whole-body soft human tissues: a review. Biomed. Mater..

[92] Shiroud Heidari B. (Jan. 2023). Natural, synthetic and commercially-available biopolymers used to regenerate tendons and ligaments. Bioact. Mater..

[93] Gan R.Z., Yang F., Zhang X., Nakmali D. (Apr. 2011). Mechanical properties of stapedial annular ligament. Med. Eng. Phys..

[94] Lin C.-Y., Sadeghi S., Bader D.A., Cortes D.H. (Feb. 2018). Ultrasound shear wave elastography of the elbow ulnar collateral ligament: reliability test and a preliminary case study in a baseball pitcher. J. Eng. Sci. Med. Diagnostics Ther..

[95] Zhang Z.J., Fu S.N. (Jun. 2013). Shear elastic modulus on patellar tendon captured from supersonic shear imaging: correlation with tangent traction modulus computed from material testing system and test-retest reliability. PLoS One.

[96] Zellers J.A., Cortes D.H., Pohlig R.T., Silbernagel K.G. (Sep. 2019). Tendon morphology and mechanical properties assessed by ultrasound show change early in recovery and potential prognostic ability for 6-month outcomes. Knee Surgery, Sport. Traumatol. Arthrosc..

[97] Martin J.A. (Apr. 2018). Gauging force by tapping tendons. Nat. Commun..

[98] Montini-Ballarin F. (Jul. 2016). Mechanical behavior of bilayered small-diameter nanofibrous structures as biomimetic vascular grafts. J. Mech. Behav. Biomed. Mater..

[99] Suh C.-M., Kim S.-H., Monson K.L., Goldsmith W. (Jul. 2003). Tensile characteristics and behavior of blood vessels from human brain in uniaxial tensile test. KSME Int. J..

[100] Li W. (Mar. 2018). Biomechanical property and modelling of venous wall. Prog. Biophys. Mol. Biol..

[101] Chen Q.Z. (Jan. 2008). Characterisation of a soft elastomer poly(glycerol sebacate) designed to match the mechanical properties of myocardial tissue. Biomaterials.

[102] Yıldırım M.A., Sanli A., Türkoğlu N., Denktaş C. (Aug. 2023). Fabrication of electrospun nanofibrous clinoptilolite doped thermoplastic polyurethane scaffolds for skeletal muscle tissue engineering. J. Appl. Polym. Sci..

[103] Reis L.A., Chiu L.L.Y., Feric N., Fu L., Radisic M. (Jan. 2016). Biomaterials in myocardial tissue engineering. J. Tissue Eng. Regen. Med..

[104] Kim H., Osaki T., Kamm R.D., Asada H.H. (Aug. 2022). Tri-culture of spatially organizing human skeletal muscle cells, endothelial cells, and fibroblasts enhances contractile force and vascular perfusion of skeletal muscle tissues. FASEB J..

[105] Zink P. (May 1972). Mechanische Eigenschaften lebensfrischer und totenstarrer menschlicher Skeletmuskelfasern und ganzer Muskeln. Z. Rechtsmed..

[106] Engler A.J., Griffin M.A., Sen S., Bönnemann C.G., Sweeney H.L., Discher D.E. (Sep. 2004). Myotubes differentiate optimally on substrates with tissue-like stiffness: pathological implications for soft or stiff microenvironments. J. Cell Biol..

[107] Gennisson J.L. (Aug. 2004). Assessment of elastic parameters of human skin using dynamic elastography. IEEE Trans. Ultrason. Ferroelectr. Freq. Control.

[108] Park S., Tao J., Sun L., Fan C.M., Chen Y. (Mar. 2019). An economic, modular, and portable skin viscoelasticity measurement device for in situ longitudinal studies. Molecules.

[109] Han F. (Feb. 2014). Woven silk fabric-reinforced silk nanofibrous scaffolds for regenerating load-bearing soft tissues. Acta Biomater..

[110] Persson N., Martinez J.G., Zhong Y., Maziz A., Jager E.W.H. (Oct. 2018). Actuating textiles: next generation of smart textiles. Adv. Mater. Technol..

[111] Sinclair R. (2015). Textiles and Fashion: Materials, Design and Technology.

[112] Hussein K.H., Park K.-M., Kang K.-S., Woo H.-M. (Oct. 2016). Biocompatibility evaluation of tissue-engineered decellularized scaffolds for biomedical application. Mater. Sci. Eng. C.

[113] Eichhorn S.J., Hearle J.W.S., Jaffe M., Kikutani T. (2009).

[114] Fourné F. (1999).

[115] Veit D. (2022).

[116] Kopf S., Åkesson D., Skrifvars M. (Jan. 2023). Textile fiber production of biopolymers–A review of spinning techniques for polyhydroxyalkanoates in biomedical applications. Polym. Rev..

[117] Tamayol A., Akbari M., Annabi N., Paul A., Khademhosseini A., Juncker D. (Sep. 2013). Fiber-based tissue engineering: progress, challenges, and opportunities. Biotechnol. Adv..

[118] Pedde R.D. (May 2017). Emerging biofabrication strategies for engineering complex tissue constructs. Adv. Mater..

[119] Naeimirad M., Krins B., Gruter G.-J.M. (Oct. 2023). A review on melt-spun biodegradable fibers. Sustainability.

[120] Hufenus R., Yan Y., Dauner M., Kikutani T. (Sep. 2020). Melt-spun fibers for textile applications. Materials.

[121] Hufenus R., Yan Y., Dauner M., Kikutani T. (Sep. 2020). Melt-spun fibers for textile applications. Materials.

[122] Rohani Shirvan A., Nouri A., Sutti A. (Dec. 2022). A perspective on the wet spinning process and its advancements in biomedical sciences. Eur. Polym. J..

[123] Ren N., Qiao A., Cui M., Huang R., Qi W., Su R. (Dec. 2023). Design and fabrication of nanocellulose-based microfibers by wet spinning. Chem. Eng. Sci..

[124] Hwang C.M. (Aug. 2009). Controlled cellular orientation on PLGA microfibers with defined diameters. Biomed. Microdevices.

[125] Xue J., Wu T., Dai Y., Xia Y. (Apr. 2019). Electrospinning and electrospun nanofibers: methods, materials, and applications. Chem. Rev..

[126] Alghoraibi I., Alomari S. (2018). Handbook of Nanofibers.

[127] Doersam A., Tsigkou O., Jones C. (Nov. 2022). A review: textile technologies for single and multi-layer tubular soft tissue engineering. Adv. Mater. Technol..

[128] Moutos F.T., Freed L.E., Guilak F. (Feb. 2007). A biomimetic three-dimensional woven composite scaffold for functional tissue engineering of cartilage. Nat. Mater..

[129] Jiang C., Wang K., Liu Y., Zhang C., Wang B. (Jul. 2021). Application of textile technology in tissue engineering: a review. Acta Biomater..

[130] Fallahi A., Khademhosseini A., Tamayol A. (Sep. 2016). Textile processes for engineering tissues with biomimetic architectures and properties. Trends Biotechnol..

[131] Akbari M. (Apr. 2016). Textile technologies and tissue engineering: a path toward organ weaving. Adv. Healthc. Mater..

[132] Loewner S. (Aug. 2022). Recent advances in melt electro writing for tissue engineering for 3D printing of microporous scaffolds for tissue engineering. Front. Bioeng. Biotechnol..

[133] Kyosev Y., Boussu F. (2022).

[134] Spencer D. (2001).

[135] Weber K.-P., Weber M.O. (2014). https://www.dfv-fachbuch.de/textiltechnik/wirkerei-strickerei,978-3-86641-299-6.html.

[136] Raz S. (1987). Melliand Textilberichte.

[137] Kyosev Y. (2014).

[138] Wu S., Wang Y., Streubel P.N., Duan B. (Oct. 2017). Living nanofiber yarn-based woven biotextiles for tendon tissue engineering using cell tri-culture and mechanical stimulation. Acta Biomater..

[139] Liberski A. (Nov. 2017). Weaving for heart valve tissue engineering. Biotechnol. Adv..

[140] Emonts C., Wienen D., Bauer B., Idrissi A., Gries T. (Nov. 2022). 3D-Braided poly-ε-caprolactone-based scaffolds for ligament tissue engineering. J. Funct. Biomater..

[141] Barber J.G., Handorf A.M., Allee T.J., Li W.-J. (Jun. 2013). Braided nanofibrous scaffold for tendon and ligament tissue engineering. Tissue Eng. Part A.

[142] Quan Q. (Apr. 2015). Preparation and characterization of braided tube reinforced polyacrylonitrile hollow fiber membranes. J. Appl. Polym. Sci..

[143] Hua T. (2020). Handbook of Fibrous Materials.

[144] Ng K.W. (Oct. 2005). Characterization of a novel bioactive poly[(lactic acid)-co-(glycolic acid)] and collagen hybrid matrix for dermal regeneration. Polym. Int..

[145] Lin M.C., Lou C.W., Lin J.Y., Lin T.A., Chen Y.S., Lin J.H. (Oct. 2018). Biodegradable polyvinyl alcohol vascular stents: structural model and mechanical and biological property evaluation. Mater. Sci. Eng. C.

[146] Wang X. (Oct. 2011). Applications of knitted mesh fabrication techniques to scaffolds for tissue engineering and regenerative medicine. J. Mech. Behav. Biomed. Mater..

[147] Weber M. (Apr. 2014). Tissue-engineered fibrin-based heart valve with a tubular leaflet design. Tissue Eng. - Part C Methods.

[148] Leong K.H., Ramakrishna S., Huang Z.M., Bibo G.A. (Mar. 2000). Potential of knitting for engineering composites - a review. Compos. Part A Appl. Sci. Manuf..

[149] Zhang X., Ma P. (Jun. 2018). Application of knitting structure textiles in medical areas. Autex Res. J..

[150] Hahn L., Zierold K., Golla A., Friese D., Rittner S. (May 2023). 3D textiles based on warp knitted fabrics: a review. Materials.

[151] Wu S. (Jun. 2022). State-of-the-art review of advanced electrospun nanofiber yarn-based textiles for biomedical applications. Appl. Mater. Today.

[152] Keirouz A., Chung M., Kwon J., Fortunato G., Radacsi N. (Jul. 2020). 2D and 3D electrospinning technologies for the fabrication of nanofibrous scaffolds for skin tissue engineering: a review. WIREs Nanomedicine and Nanobiotechnology.

[153] Balguid A., Mol A., van Marion M.H., Bank R.A., Bouten C.V.C., Baaijens F.P.T. (Feb. 2009). Tailoring fiber diameter in electrospun poly(ɛ-caprolactone) scaffolds for optimal cellular infiltration in cardiovascular tissue engineering. Tissue Eng. Part A.

[154] Hollister S.J., Maddox R.D., Taboas J.M. (Oct. 2002). Optimal design and fabrication of scaffolds to mimic tissue properties and satisfy biological constraints. Biomaterials.

[155] Lee Y.H. (Jun. 2005). Electrospun dual-porosity structure and biodegradation morphology of Montmorillonite reinforced PLLA nanocomposite scaffolds. Biomaterials.

[156] Chong E.J. (May 2007). Evaluation of electrospun PCL/gelatin nanofibrous scaffold for wound healing and layered dermal reconstitution. Acta Biomater..

[157] Zhu X., Cui W., Li X., Jin Y. (Jul. 2008). Electrospun fibrous mats with high porosity as potential scaffolds for skin tissue engineering. Biomacromolecules.

[158] Shao W. (Oct. 2016). A biomimetic multilayer nanofiber fabric fabricated by electrospinning and textile technology from polylactic acid and Tussah silk fibroin as a scaffold for bone tissue engineering. Mater. Sci. Eng. C.

[159] Vaquette C. (Sep. 2010). Aligned poly(L-lactic-co-e-caprolactone) electrospun microfibers and knitted structure: a novel composite scaffold for ligament tissue engineering. J. Biomed. Mater. Res., Part A.

[160] Deng D. (Oct. 2014). Repair of Achilles tendon defect with autologous ASCs engineered tendon in a rabbit model. Biomaterials.

[161] Sharifi-Aghdam M., Faridi-Majidi R., Derakhshan M.A., Chegeni A., Azami M. (Jul. 2017). Preparation of collagen/polyurethane/knitted silk as a composite scaffold for tendon tissue engineering. Proc. Inst. Mech. Eng. Part H J. Eng. Med..

[162] Vaquette C., Sudheesh Kumar P.T., Petcu E.B., Ivanovski S. (Jan. 2018). Combining electrospinning and cell sheet technology for the development of a multiscale tissue engineered ligament construct (TELC). J. Biomed. Mater. Res. Part B Appl. Biomater..

[163] Zhang Y. (Apr. 2017). A compliant and biomimetic three-layered vascular graft for small blood vessels. Biofabrication.

[164] Rothrauff B.B., Lauro B.B., Yang G., Debski R.E., Musahl V., Tuan R.S. (May 2017). Braided and stacked electrospun nanofibrous scaffolds for tendon and ligament tissue engineering. Tissue Eng. - Part A.

[165] Mi H.-Y. (May 2019). Fabrication of triple-layered vascular grafts composed of silk fibers, polyacrylamide hydrogel, and polyurethane nanofibers with biomimetic mechanical properties. Mater. Sci. Eng. C.

[166] He J., Hao G., Meng Z., Cao Y., Li D. (Jul. 2022). Expanding melt‐based electrohydrodynamic printing of highly‐ordered microfibrous architectures to Cm‐height via in situ charge neutralization. Adv. Mater. Technol..

[167] Castilho M. (Sep. 2017). Melt electrospinning writing of poly‐hydroxymethylglycolide‐ co ‐ε‐Caprolactone‐Based scaffolds for cardiac tissue engineering. Adv. Healthc. Mater..

[168] Eichholz K.F., Hoey D.A. (Jul. 2018). Mediating human stem cell behaviour via defined fibrous architectures by melt electrospinning writing. Acta Biomater..

[169] Bosworth L.A., Lanaro M., O'Loughlin D.A., D’Sa R.A., Woodruff M.A., Williams R.L. (Jan. 2022). Melt electro-written scaffolds with box-architecture support orthogonally oriented collagen. Biofabrication.

[170] Hoffmann L., Jung S., Bettermann I. (2024). Elastic Yarns and Textiles.

[171] Laurent C.P., Durville D., Mainard D., Ganghoffer J.-F., Rahouadj R. (Aug. 2012). A multilayer braided scaffold for Anterior Cruciate Ligament: mechanical modeling at the fiber scale. J. Mech. Behav. Biomed. Mater..

[172] Afghah F., Dikyol C., Altunbek M., Koc B. (Aug. 2019). Biomimicry in bio-manufacturing: developments in melt electrospinning writing technology towards hybrid biomanufacturing. Appl. Sci..

[173] Liao I., Moutos F.T., Estes B.T., Zhao X., Guilak F. (Dec. 2013). Composite three‐dimensional woven scaffolds with interpenetrating network hydrogels to create functional synthetic articular cartilage. Adv. Funct. Mater..

[174] Gu X., Ding F., Williams D.F. (Aug. 2014). Neural tissue engineering options for peripheral nerve regeneration. Biomaterials.

[175] Boni R., Ali A., Shavandi A., Clarkson A.N. (Dec. 2018). Current and novel polymeric biomaterials for neural tissue engineering. J. Biomed. Sci..

[176] Li Z. (Jul. 2023). Nervous tract-bioinspired multi-nanoyarn model system regulating neural differentiation and its transcriptional architecture at single-cell resolution. Biomaterials.

[177] Lu M.-C. (May 2009). Evaluation of a multi-layer microbraided polylactic acid fiber-reinforced conduit for peripheral nerve regeneration. J. Mater. Sci. Mater. Med..

[178] Wang A. (Oct. 2006). Porous chitosan tubular scaffolds with knitted outer wall and controllable inner structure for nerve tissue engineering. J. Biomed. Mater. Res., Part A.

[179] Politikou O. (Mar. 2024). Digital nerve reconstruction with a new composite silk fibroin nerve conduit. J. Peripher. Nerv. Syst..

[180] Goodarzi P. (2018). Tissue engineered skin substitutes. Adv. Exp. Med. Biol..

[181] Vig K. (Apr. 2017). Advances in skin regeneration using tissue engineering. Int. J. Mol. Sci..

[182] Ng K.W., Hutmacher D.W. (Sep. 2006). Reduced contraction of skin equivalent engineered using cell sheets cultured in 3D matrices. Biomaterials.

[183] Chen G., Sato T., Ohgushi H., Ushida T., Tateishi T., Tanaka J. (May 2005). Culturing of skin fibroblasts in a thin PLGA–collagen hybrid mesh. Biomaterials.

[184] Afrashi M., Semnani D., Hashemibeni B., Shokrgozar M.A. (Mar. 2024). Degradable and biocompatible nanofibrous scaffold incorporating a natural cell culture medium for skin tissue engineering. Phys. Scr..

[185] Dias J.R., Granja P.L., Bártolo P.J. (Dec. 2016). Advances in electrospun skin substitutes. Prog. Mater. Sci..

[186] Shanti R.M. (Nov. 2008). In vitro adipose tissue engineering using an electrospun nanofibrous scaffold. Ann. Plast. Surg..

[187] Li W.J., Cooper J.A., Mauck R.L., Tuan R.S. (Jul. 2006). Fabrication and characterization of six electrospun poly(α-hydroxy ester)-based fibrous scaffolds for tissue engineering applications. Acta Biomater..

[188] Schäfer B. (Aug. 2020). Warp-Knitted spacer fabrics: a versatile platform to generate fiber-reinforced hydrogels for 3D tissue engineering. Materials.

[189] Zhang Q. (Jan. 2025). Bamboo-like’ strong and tough sodium alginate/polyacrylate hydrogel fiber with directional controlled release for wound healing promotion. Carbohydr. Polym..

[190] Zhang S. (May 2023). Large-scale manufacturing of soluble hemostatic spacer dressing with excellent mechanical and comfortable properties. Mater. Des..

[191] Durko A.P., Yacoub M.H., Kluin J. (Apr. 2020). Tissue engineered materials in cardiovascular surgery: the surgeon's perspective. Front. Cardiovasc. Med..

[192] Fioretta E.S., von Boehmer L., Motta S.E., Lintas V., Hoerstrup S.P., Emmert M.Y. (Mar. 2019). Cardiovascular tissue engineering: from basic science to clinical application. Exp. Gerontol..

[193] Dogan A., Eser Elcin A., Murat Elcin Y. (Mar. 2017). Translational applications of tissue engineering in cardiovascular medicine. Curr. Pharm. Des..

[194] Hu Q. (Oct. 2020). Fabrication of multilayer tubular scaffolds with aligned nanofibers to guide the growth of endothelial cells. J. Biomater. Appl..

[195] Fukunishi T. (Apr. 2019). Formation of neoarteries with optimal remodeling using rapidly degrading textile vascular grafts. Tissue Eng. Part A.

[196] Wei Y., Sun D., yi H., Wang J. (Jun. 2014). Characterization of a PEG-DE cross-linked tubular silk scaffold. Text. Res. J..

[197] Liu Z., Zheng Z., Chen K., Li Y., Wang X., Li G. (Aug. 2019). A heparin-functionalized woven stent graft for endovascular exclusion. Colloids Surfaces B Biointerfaces.

[198] Rama E. (Dec. 2024). In vitro and in vivo evaluation of biohybrid tissue-engineered vascular grafts with transformative 1H/19F MRI traceable scaffolds. Biomaterials.

[199] Yousefi A., Vaesken A., Amri A., Dasi L.P., Heim F. (Feb. 2017). Heart valves from polyester fibers vs. Biological tissue: comparative study in vitro. Ann. Biomed. Eng..

[200] Ross R., Salein J., Menne M., Mela P., Jockenhoevel S., Gries T. (Jan. 2013). Textile reinforcement in fibrin-based tissue engineerd heart valves. Biomed. Eng./Biomed. Tech..

[201] Xu C. (Jun. 2024). Melt-electrowriting-enabled anisotropic scaffolds loaded with valve interstitial cells for heart valve tissue Engineering. J. Nanobiotechnology.

[202] Boublik J. (Jul. 2005). Mechanical properties and remodeling of hybrid cardiac constructs made from heart cells, fibrin, and biodegradable, elastomeric knitted fabric. Tissue Eng..

[203] Gupta M.K. (Dec. 2011). Combinatorial polymer electrospun matrices promote physiologically-relevant cardiomyogenic stem cell differentiation. PLoS One.

[204] O'Leary C., Gilbert J.L., O'Dea S., O'Brien F.J., Cryan S.A. (Aug. 2015). Respiratory tissue engineering: current status and opportunities for the future. Tissue Eng. - Part B Rev..

[205] Law J.X., Liau L.L., Aminuddin B.S., Ruszymah B.H.I. (Dec. 2016). Tissue-engineered trachea: a review. Int. J. Pediatr. Otorhinolaryngol..

[206] Wu T. (Dec. 2017). Application of a bilayer tubular scaffold based on electrospun poly(l-lactide-co-caprolactone)/collagen fibers and yarns for tracheal tissue engineering. J. Mater. Chem. B.

[207] Tatekawa Y., Kawazoe N., Chen G., Shirasaki Y., Komuro H., Kaneko M. (Jun. 2010). Tracheal defect repair using a PLGA–collagen hybrid scaffold reinforced by a copolymer stent with bFGF-impregnated gelatin hydrogel. Pediatr. Surg. Int..

[208] Horst M. (Jul. 2013). Engineering functional bladder tissues. J. Tissue Eng. Regen. Med..

[209] Mahfouz W., Elsalmy S., Corcos J., Fayed A.S. (Jun. 2013). Fundamentals of bladder tissue engineering. African J. Urol..

[210] Lam Van Ba O., Aharony S., Loutochin O., Corcos J. (Mar. 2015). Bladder tissue engineering: a literature review. Adv. Drug Deliv. Rev..

[211] Drewa T., Adamowicz J., Sharma A. (Oct. 2012). Tissue engineering for the oncologic urinary bladder. Nat. Rev. Urol..

[212] Siegel R.L., Giaquinto A.N., Jemal A. (Jan. 2024). Cancer statistics, 2024. CA Cancer J. Clin..

[213] Atala A., Bauer S.B., Soker S., Yoo J.J., Retik A.B. (Apr. 2006). Tissue-engineered autologous bladders for patients needing cystoplasty. Lancet.

[214] Surgical Mesh Used for Hernia Repair” FDA Administration. Accessed: May 06, 2024. [Online]. Available: https://www.fda.gov/medical-devices/implants-and-prosthetics/surgical-mesh-used-hernia-repair.

[215] Khademolqorani S., Tavanai H., Ajalloueian F. (Jun. 2021). Mechanical properties of silk plain‐weft knitted scaffolds for bladder tissue engineering applications. Polym. Adv. Technol..

[216] Feng C., Liu C., Liu S., Wang Z., Yu K., Zeng X. (Sep. 2019). Electrospun nanofibers with core-shell structure for treatment of bladder regeneration. Tissue Eng. - Part A.

[217] Stankus J.J., Freytes D.O., Badylak S.F., Wagner W.R. (May 2008). Hybrid nanofibrous scaffolds from electrospinning of a synthetic biodegradable elastomer and urinary bladder matrix. J. Biomater. Sci. Polym. Ed..

[218] Seifi Z., Khazaei M., Cheraghali D., Rezakhani L. (Jun. 2024). Decellularized tissues as platforms for digestive system cancer models. Heliyon.

[219] Yagi H., Soto-Gutierrez A., Kitagawa Y. (Jun. 2013). Whole-organ re-engineering: a regenerative medicine approach in digestive surgery for organ replacement. Surg. Today.

[220] Chaturvedi R.R. (May 2015). Patterning vascular networks in vivo for tissue engineering applications. Tissue Eng. - Part C Methods.

[221] Yekrang J., Semnani D., Seyghlani A.Z. (Jun. 2022). Simulation and characterization of the mechanical properties of knitted esophageal stents using finite element and mathematical models. J. Ind. Text..

[222] Karamuk E., Mayer J., Wintermantel E., Akaike T. (Sep. 1999). Partially degradable film/fabric composites: textile scaffolds for liver cell culture. Artif. Organs.

[223] Risbud M.V., Karamuk E., Moser R., Mayer J. (May 2002). Hydrogel-coated textile scaffolds as three-dimensional growth support for human umbilical vein endothelial cells (HUVECs): possibilities as coculture system in liver tissue engineering. Cell Transplant..

[224] Kuppan P., Sethuraman S., Krishnan U.M. (Mar. 2016). Fabrication and investigation of nanofibrous matrices as esophageal tissue scaffolds using human non-keratinized, stratified, squamous epithelial cells. RSC Adv..

[225] Ghahremanzadeh F., Alihosseini F., Semnani D. (Mar. 2021). Investigation and comparison of new galactosylation methods on PCL/chitosan scaffolds for enhanced liver tissue engineering. Int. J. Biol. Macromol..

[226] Neuhäusler A. (Jan. 2025). Electrospun microfibers to enhance nutrient supply in bioinks and 3D-bioprinted tissue precursors. Biofabrication.

[227] Zhang L. (Mar. 2021). Systematic review of silk scaffolds in musculoskeletal tissue engineering applications in the recent decade. ACS Biomater. Sci. Eng..

[228] Zhang X., Wang D., Mak K.-L.K., Tuan R.S., Ker D.F.E. (Aug. 2021). Engineering musculoskeletal grafts for multi-tissue unit repair: lessons from developmental biology and wound healing. Front. Physiol..

[229] Del Bakhshayesh A.R. (Dec. 2019). An overview of advanced biocompatible and biomimetic materials for creation of replacement structures in the musculoskeletal systems: focusing on cartilage tissue engineering. J. Biol. Eng..

[230] Tan G., Zhou Y., Sooriyaarachchi D. (2021). Musculoskeletal tissue engineering using fibrous biomaterials. Methods Mol. Biol..

[231] Vigodarzere G.C., Mantero S. (Sep. 2014). Skeletal muscle tissue engineering: strategies for volume tric constructs. Front. Physiol..

[232] Pien N. (Apr. 2023). Tissue engineering of skeletal muscle, tendons and nerves: a review of manufacturing strategies to meet structural and functional requirements. Appl. Mater. Today.

[233] Thangadurai M., Ajith A., Budharaju H., Sethuraman S., Sundaramurthi D. (Nov. 2022). Advances in electrospinning and 3D bioprinting strategies to enhance functional regeneration of skeletal muscle tissue. Biomater. Adv..

[234] Zhang Y., Zhang Z., Wang Y., Su Y., Chen M. (Nov. 2020). 3D myotube guidance on hierarchically organized anisotropic and conductive fibers for skeletal muscle tissue engineering. Mater. Sci. Eng. C.

[235] Deshpande M.V., West A.J., Bernacki S.H., Luan K., King M.W. (2020). Poly(ε-Caprolactone) resorbable auxetic designed knitted scaffolds for craniofacial skeletal muscle regeneration. Bioengineering.

[236] Woo S.L.Y., Debski R.E., Zeminski J., Abramowitch S.D., Chan Saw S.S., Fenwick J.A. (Aug. 2000). Injury and repair of ligaments and tendons. Annu. Rev. Biomed. Eng..

[237] Shiroud Heidari B., Ruan R., De-Juan-Pardo E.M., Zheng M., Doyle B. (Feb. 2021). Biofabrication and signaling strategies for tendon/ligament interfacial tissue engineering. ACS Biomater. Sci. Eng..

[238] Rodrigues M.T., Reis R.L., Gomes M.E. (Sep. 2013). Engineering tendon and ligament tissues: present developments towards successful clinical products. J. Tissue Eng. Regen. Med..

[239] Cooper J.A., Lu H.H., Ko F.K., Freeman J.W., Laurencin C.T. (May 2005). Fiber-based tissue-engineered scaffold for ligament replacement: design considerations and in vitro evaluation. Biomaterials.

[240] Freeman J.W., Woods M.D., Laurencin C.T. (Jan. 2007). Tissue engineering of the anterior cruciate ligament using a braid–twist scaffold design. J. Biomech..

[241] Emonts C. (Aug. 2024). Mechanical, biological and in vitro degradation investigation of braided scaffolds for tendon and ligament tissue engineering based on different polycaprolactone materials with chitosan-graft-PCL surface modification. Polymers.

[242] Sahoo S., Cho-Hong J.G., Siew-Lok T. (Sep. 2007). Development of hybrid polymer scaffolds for potential applications in ligament and tendon tissue engineering. Biomed. Mater..

[243] Sahoo S., Toh S.L., Goh J.C.H. (Apr. 2010). A bFGF-releasing silk/PLGA-based biohybrid scaffold for ligament/tendon tissue engineering using mesenchymal progenitor cells. Biomaterials.

[244] Hoshi K., Fujihara Y., Yamawaki T., Harai M., Asawa Y., Hikita A. (Apr. 2018). Biological aspects of tissue-engineered cartilage. Histochem. Cell Biol..

[245] He Y.-J., Lin S., Ao Q. (Jan. 2019). Research progress of tissue-engineered cartilage in repairing cartilage defects. Sci. Adv. Mater..

[246] Chung C., Burdick J.A. (Jan. 2008). Engineering cartilage tissue. Adv. Drug Deliv. Rev..

[247] Ahn H., Kim K.J., Park S.Y., Huh J.E., Kim H.J., Yu W.-R. (Jun. 2014). 3D braid scaffolds for regeneration of articular cartilage. J. Mech. Behav. Biomed. Mater..

[248] Dai W., Yao Z., Dong J., Kawazoe N., Zhang C., Chen G. (May 2013). Cartilage tissue engineering with controllable shape using a poly(lactic- co -glycolic acid)/collagen hybrid scaffold. J. Bioact. Compat Polym..

[249] Xue N. (Jul. 2022). Bone tissue engineering in the treatment of bone defects. Pharmaceuticals.

[250] Dec P., Modrzejewski A., Pawlik A. (Dec. 2022). Existing and novel biomaterials for bone tissue engineering. Int. J. Mol. Sci..

[251] Jakob F. (Jun. 2013). Bone tissue engineering in osteoporosis. Maturitas.

[252] Wu S., Liu X., Yeung K.W.K., Liu C., Yang X. (Jun. 2014). Biomimetic porous scaffolds for bone tissue engineering. Mater. Sci. Eng. R Reports.

[253] Xue J. (Jul. 2022). A biodegradable 3D woven magnesium-based scaffold for orthopedic implants. Biofabrication.

[254] Rentsch C. (Jan. 2010). Evaluation of the osteogenic potential and vascularization of 3D poly(3)hydroxybutyrate scaffolds subcutaneously implanted in nude rats. J. Biomed. Mater. Res., Part A.

[255] Ribeiro V.P. (Apr. 2017). Silk-based anisotropical 3D biotextiles for bone regeneration. Biomaterials.

[256] Pandala N., Haywood S., LaScola M.A., Day A., Leckron J., Lavik E. (Nov. 2020). Screen printing to create 3D tissue models. ACS Appl. Bio Mater..

[257] Albrecht F.B., Ahlfeld T., Klatt A., Heine S., Gelinsky M., Kluger P.J. (May 2024). Biofabrication's contribution to the evolution of cultured meat. Adv. Healthc. Mater..

[258] Halbrecht A., Kinsbursky M., Poranne R., Sterman Y. (Mar. 2023). 3D printed spacer fabrics. Addit. Manuf..

[259] Ge M., Chen F., Chen C., Cong H., Dong Z., Ma P. (Sep. 2024). Large-scale production of a ‘skin-like’ self-pumping fabric for personal sweat management. Chem. Eng. J..

[260] Xing T. (Oct. 2023). Silk-based flexible electronics and smart wearable Textiles: progress and beyond. Chem. Eng. J..

[261] Wang W. (May 2023).

[262] Liu K. (Jun. 2024). Conductive and alignment-optimized porous fiber conduits with electrical stimulation for peripheral nerve regeneration. Mater. Today Bio.

[263] Li Y., Zhang X., Ying B.A. (Jun. 2019). On textile biomedical engineering. Sci. China Technol. Sci..

[264] Harimurti S. (Jun. 2022). Review—human-body powered biosensing textiles: body-power generating wearables based on textiles for human biomonitoring. J. Electrochem. Soc..

[265] Kar A., Ahamad N., Dewani M., Awasthi L., Patil R., Banerjee R. (Apr. 2022). Wearable and implantable devices for drug delivery: applications and challenges. Biomaterials.

